# Dynamic Allostery Mediated by a Conserved Tryptophan in the Tec Family Kinases

**DOI:** 10.1371/journal.pcbi.1004826

**Published:** 2016-03-24

**Authors:** Nikita Chopra, Thomas E. Wales, Raji E. Joseph, Scott E. Boyken, John R. Engen, Robert L. Jernigan, Amy H. Andreotti

**Affiliations:** 1 Roy J. Carver Department of Biochemistry, Biophysics and Molecular Biology, Iowa State University, Ames, Iowa, United States of America; 2 Department of Chemistry and Chemical Biology, Northeastern University, Boston, Massachusetts, United States of America; 3 Department of Biochemistry, University of Washington, Seattle, Washington, United States of America; Bogazici University, TURKEY

## Abstract

Bruton’s tyrosine kinase (Btk) is a Tec family non-receptor tyrosine kinase that plays a critical role in immune signaling and is associated with the immunological disorder X-linked agammaglobulinemia (XLA). Our previous findings showed that the Tec kinases are allosterically activated by the adjacent N-terminal linker. A single tryptophan residue in the N-terminal 17-residue linker mediates allosteric activation, and its mutation to alanine leads to the complete loss of activity. Guided by hydrogen/deuterium exchange mass spectrometry results, we have employed Molecular Dynamics simulations, Principal Component Analysis, Community Analysis and measures of node centrality to understand the details of how a single tryptophan mediates allostery in Btk. A specific tryptophan side chain rotamer promotes the functional dynamic allostery by inducing coordinated motions that spread across the kinase domain. Either a shift in the rotamer population, or a loss of the tryptophan side chain by mutation, drastically changes the coordinated motions and dynamically isolates catalytically important regions of the kinase domain. This work also identifies a new set of residues in the Btk kinase domain with high node centrality values indicating their importance in transmission of dynamics essential for kinase activation. Structurally, these node residues appear in both lobes of the kinase domain. In the N-lobe, high centrality residues wrap around the ATP binding pocket connecting previously described Catalytic-spine residues. In the C-lobe, two high centrality node residues connect the base of the R- and C-spines on the αF-helix. We suggest that the bridging residues that connect the catalytic and regulatory architecture within the kinase domain may be a crucial element in transmitting information about regulatory spine assembly to the catalytic machinery of the catalytic spine and active site.

## Author Summary

Bruton’s tyrosine kinase (Btk) belongs to the Tec family of protein tyrosine kinases, and plays a crucial role in the signaling pathway in B-cells. Alteration of Btk activity results in the serious immunological disorder, X-linked agammaglobulinemia. Btk is a multi-domain protein and the activity of the kinase domain is regulated by the adjacent non-catalytic domains, which mediate their effect by means of a conserved tryptophan residue. In this work, we have investigated the mechanism of regulation by this tryptophan residue, W395, in the linker preceding the Btk kinase domain. Using hydrogen-deuterium exchange mass spectrometry and molecular dynamics simulations we identify structural elements within the kinase domain that are required for function by transmitting the allosteric effects of W395. Molecular Dynamics simulations further guided us to delineate the kinase domain into dynamically correlated sets of residues using community analysis, thereby identifying the important communication nodes that connect the various elements of the kinase domain required for function. The analyses performed indicate clearly how the W395A mutant changes the communication pathway required for function.

## Introduction

**Fig 1:**
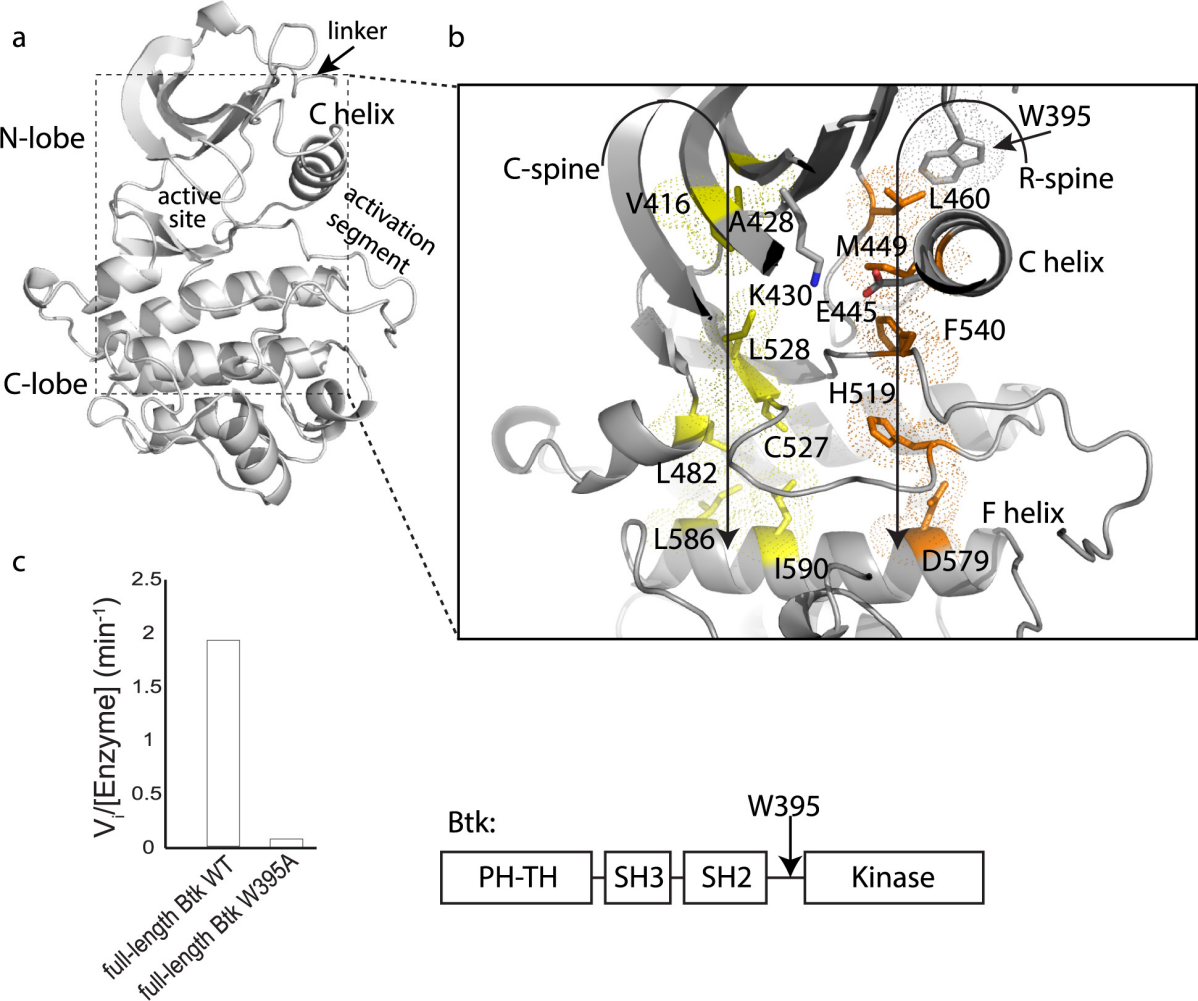
W395 is required for Btk activity. (a) Btk linker-kinase domain structure (PDB ID: 3K54) showing the linker, N- and C-lobes, active site, and activation segment. (b) Key regulatory elements in the Btk linker-kinase domain are the R- and C-spines, orange and yellow, respectively. ATP completes the C-spine structure in the N-lobe but is omitted here for clarity. W395 is shown above the αC-helix and the residues in the conserved salt bridge, K430 and E445 are labeled. The C- and R-spines are supported by the αF-helix in the C-lobe of the kinase domain. (c) Initial velocity measurements comparing the activity of full-length Btk (domain structure is shown to the right) to full-length Btk (W395A) using the poly (4:1, Glu:Tyr) peptide substrate [11].

Kinase domains consist of a bi-lobal structure, the N- and C-lobes, which together create the catalytic site to transfer a phosphate from ATP to a substrate hydroxyl (Fig 1A) [1, 2]. Transition between active and inactive kinase structures involves concerted motions of specific regions of secondary structure. For example, the αC-helix in the N-lobe adopts a ‘C-out’ conformation in the inactive state and shifts to a ‘C-in’ conformation when the kinase is activated. This transition is triggered by phosphorylation of specific residues in the activation loop that cause a switch in a number of electrostatic interactions. Moreover, the αF-helix in the C-lobe supports two separate regulatory motifs (the Catalytic (C)-spine and Regulatory (R)-spine) [3–5] (Fig 1B). The residues that make up these spines are conserved and their proper assembly is required for kinase activation. Identification of these spines has provided a model for kinase activation that explains how phosphorylation at regulatory sites on the activation loop triggers subsequent conformational rearrangements that stabilize the active kinase domain [3–5].

The Tec family of non-receptor tyrosine kinases, Btk, Itk, Tec, Bmx and Rlk, are regulators of immune cell function [6, 7]. Tec kinase mutations have been linked to immunodeficiencies and lymphoproliferative diseases [8, 9]. For example, genetic defects leading to single amino acid changes in Btk cause X-linked agammaglobulinemia or XLA, a condition characterized by a lack of mature B cells and hence a complete lack of circulating antibodies. A clear understanding of Tec family regulation is a critical step needed prior to developing improved immunotherapies.

Consistent with the R-spine model, we have found that individual mutation of each of the R-spine residues in Itk disrupts catalytic activity [10]. However, we have also found that the isolated Itk and Btk kinase domains have reduced activities compared to the corresponding full-length enzymes [11]. This suggests that even when these kinase domains are requisitely phosphorylated at the regulatory tyrosine, the active conformation (containing an assembled R-spine) is not sufficiently stable. Indeed, crystal structures of the phosphorylated Itk kinase domain reveal a disassembled R-spine [12].

Stabilization of the catalytically competent conformation must require specific contacts between residues outside of the kinase domain and the kinase domain itself to overcome the innate conformational preference of the isolated Tec family kinase domain for the inactive state [11, 13]. Mutagenesis experiments identified a conserved tryptophan (W395 in Btk) in the region preceding the kinase domain that is absolutely required for kinase activity (Fig 1C). This tryptophan plays the opposite role in the Src family kinases where it instead functions as a wedge, preventing the inward motion of αC-helix to the active ‘C-in’ state, thus stabilizing the inactive conformation of the kinase domain. Quite unlike the Tec family kinases (Fig 1C), mutation of this conserved tryptophan in Src kinases relieves the steric hindrance imposed by its side-chain resulting in a shift in the conformational equilibrium to the active state [11, 14].

In our earlier work, we proposed that the tryptophan ‘caps’ the top of the regulatory spine of the Tec family kinases providing essential contacts that stabilize the assembled R-spine structure [11]. Here, we combine results from hydrogen-deuterium exchange mass spectrometry (HDXMS) and Molecular Dynamic (MD) simulations to develop a more detailed mechanistic understanding for how the linker tryptophan drives the conformational equilibrium and dynamic sampling of the Btk kinase domain toward the active state.

## Results

### W395 exerts a positive allosteric effect within the linker-kinase domain fragment of Btk

**Fig 2:**
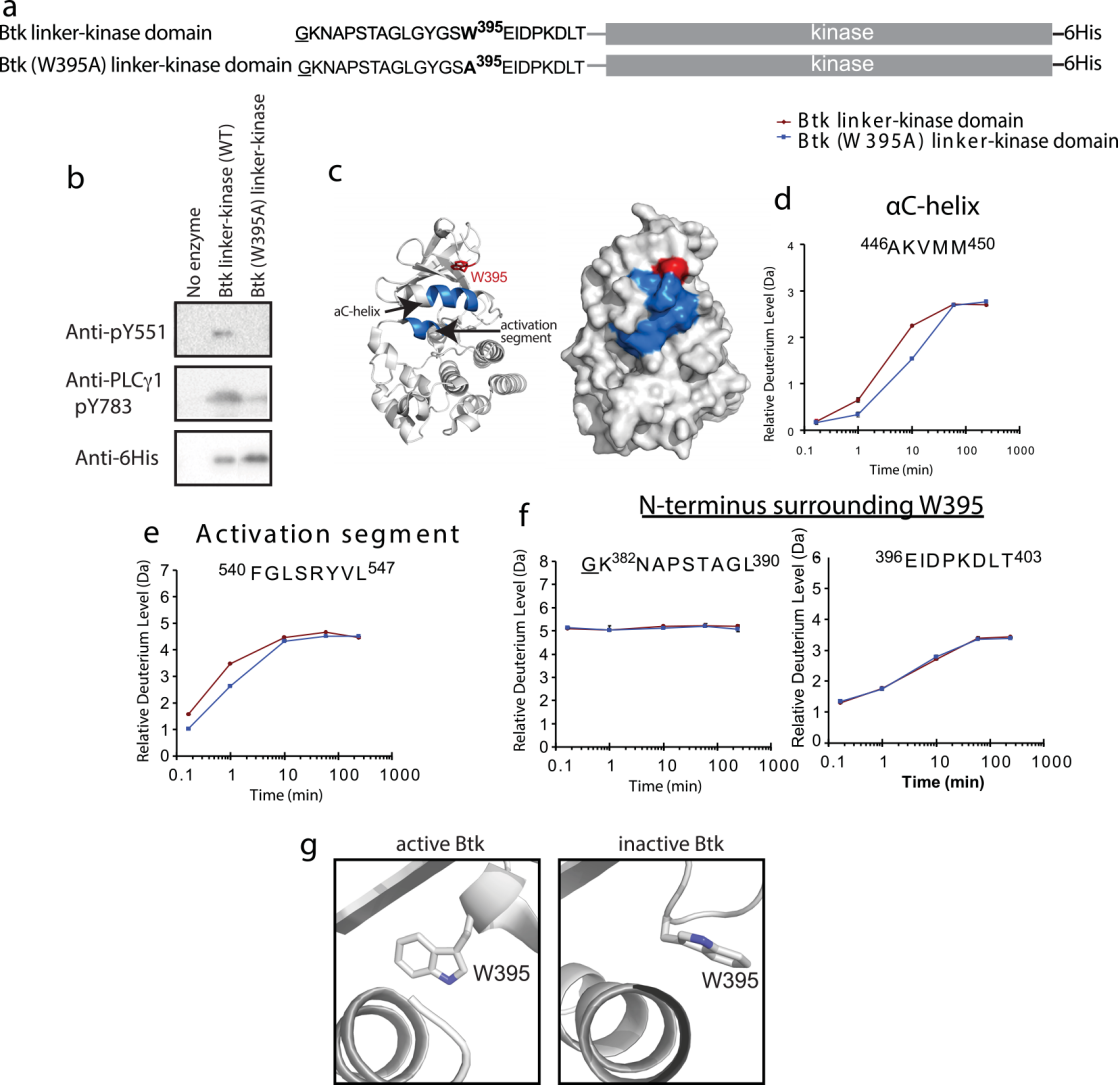
HDXMS reveals greater conformational sampling for active Btk linker-kinase domain. (a) Constructs used for activity assays and HDXMS study. The underlined residue is vector derived and not part of the sequence of the Btk kinase domain. Both Btk linker-kinase and Btk (W395A) linker-kinase carried a hexahistidine tag (6His) at the C-terminus. (b) Western blot assay to probe phosphorylation of the Btk activation loop Y551 and Y783 in the PLCγ1 substrate. Anti-His antibody recognizes the 6-His tag on the Btk constructs and is used to detect the amount of Btk enzyme in each reaction. (c) Differences greater than 0.7 Da at any one of the five time points between 10 seconds and 4 hours in the hydrogen-deuterium exchange experiment are mapped onto two depictions of the Btk linker-kinase domain (PDB ID: 3GEN) and colored in blue. The side chain of W395 is red and labeled. (d-e) Deuterium exchange for peptides derived from the αC-helix and activation segment in Btk linker-kinase (red) and Btk (W395A) linker-kinase (blue). (f) Deuterium exchange for peptides derived from the linker and N-terminal region of Btk linker-kinase (red) and Btk (W395A) linker-kinase (blue). Complete HDX data is provided in Fig A in S1 Text. (g) Side-chain rotamer conformations of W395 in the structures of active (3K54) and inactive (3GEN) Btk linker-kinase.

To probe the role of W395 in stabilizing the active form of the Btk kinase domain, we first assessed the effect of the W395A mutation within the fragment of Btk containing the kinase domain and N-terminal linker, residues 382–659 (Fig 2A). The wild type sequence is referred to as the Btk linker-kinase and was compared throughout this work to the same Btk fragment bearing the single tryptophan to alanine mutation: Btk (W395A) linker-kinase. The loss of activity observed upon mutation of W395 to alanine in the context of the Btk linker-kinase fragment (Fig 2B) mirrored that of full length Btk (Fig 1C), making this fragment a reasonable model for studies to investigate the role of W395 in controlling Btk catalytic activity.

### Deuterium exchange reveals dynamic differences between wild type and mutant Btk linker-kinase

HDXMS allows detection of backbone amide hydrogens for each amino acid in the protein (except proline) permitting direct comparison of the combined effects of solvent accessibility and hydrogen-bonding of amide N-H groups between two proteins. Btk linker-kinase and Btk (W395A) linker-kinase were subjected to identical experimental conditions allowing for exchange of deuterium with the labile amide hydrogens [15]. The H/D exchange reaction was quenched and the proteins were proteolyzed to yield peptides for analysis by mass spectrometry. Differences in deuterium exchange between wild type and the W395A mutant of Btk linker-kinase localize to the αC-helix and the N-terminal region of the activation segment (Fig 2C) with no significant differences throughout the rest of the kinase domain (see Fig A in S1 Text). For the peptides derived from the αC-helix and activation segment, deuterium uptake was greater for the wild type Btk linker-kinase protein compared to the W395A mutant (Fig 2D and 2E). This observation is consistent with previous H/D exchange data showing that a more active kinase undergoes greater conformational sampling and thus greater exchange with bulk solvent [16, 17].

HDXMS data for wild type Btk linker-kinase and Btk (W395A) linker-kinase also suggest that the linker and N-terminal region of the kinase domain are not perturbed by the W395A mutation (Fig 2F). The sequence adjacent to W395 (E^396^IDPKDLT^403^) was protected from exchange at early time points suggesting that this region associates with the N-lobe regardless of the presence or absence of the tryptophan side chain. Crystal structures of active and inactive Btk kinase domain containing the linker region indicate a single structural difference in this region; the W395 side chain adopts distinct chi1 rotamers in each structure (Fig 2G). These data suggest that in one rotameric configuration, W395 might regulate catalytic activity by increasing the dynamic motions of the adjacent kinase domain and specifically promoting more frequent conformational sampling of the αC-helix and activation segment near the active conformation, while the other rotamer moves the W395 side-chain away from the kinase domain thereby altering the environment of the αC-helix and thus the kinase domain dynamics. Mutation of W395 to alanine mimics the inactive state by removing the large side chain from the proximity of the αC-helix. To gain further insight into the allosteric role of Btk W395, we next compared all-atom MD simulations between the active Btk linker-kinase domain fragment and the inactive mutant Btk (W395A) linker-kinase domain.

### Molecular Dynamics simulations capture the activating effect of W395

**Fig 3:**
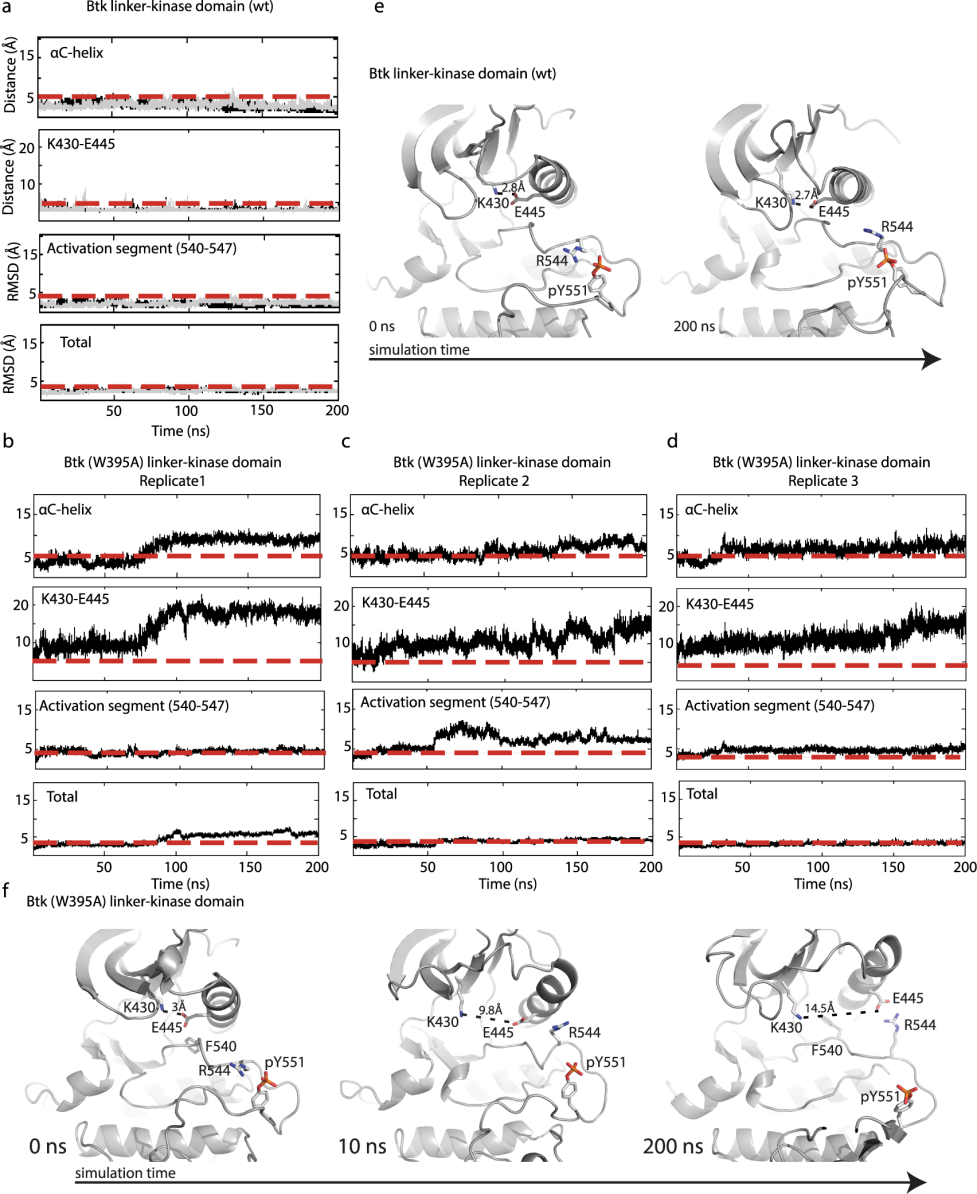
MD simulations of Btk linker-kinase and Btk (W395A) linker-kinase domains. Two replicate (200 nanosecond (ns) each) equilibrium simulations (black/light grey traces) are shown for Btk linker-kinase ((a), superimposed) and three 200 ns replicates of (W395A) linker-kinase domain are shown in (b,c,d). RMSD from the starting structure is reported for the backbone atoms (total), activation segment (540–547) and αC-helix (439–451). The distance (Å) between the side-chains of K430 and E445 is shown over the course of the simulations. The red dashed lines in each plot are included for ease of comparison between mutant and wild type trajectories. (e,f) Snapshots of structures from the wild type Btk linker-kinase simulation at 0 and 200 ns (e) and from the Btk (W395A) linker-kinase simulation at 0, 10 and 200 ns (f). The wild type Btk linker-kinase domain retains the active conformation throughout the 200 ns simulation. The K430/E445 salt bridge distance is indicated and the interaction between R544 and pY551 is evident at the beginning and end of the simulation. Btk (W395A) linker-kinase domain starts in the active conformation but moves toward the inactive state as early as 10 ns. Further transition to the inactive conformation (‘C-out’) is observed as the simulation progresses and at 200 ns the K430/E445 distance is 14.5Å and R544 contacts the side chain of E445 rather than pY551. F540 is shown at 0 and 200 ns in the Btk (W395A) linker-kinase structures to illustrate the shift from the active DFG conformation to inactive.

MD simulations are widely used to investigate the differences in conformational dynamics of mutant and wild type protein structures [14, 18, 19]. The application of MD simulations in this study aims to capture the effect of a single point mutation on the active state of the Btk linker-kinase domain. PDB ID 3K54 [20] was used to derive the starting active conformation of Btk linker-kinase domain. A detailed description of model building and mutation of W395 to alanine is provided in Methods. Three replicates of MD simulations were carried out for 200 ns for each protein model and, guided by the HDX data, we compared the RMSD changes for the αC-helix and the activation segment (Fig 3). The αC-helix remained in the active “C-in” conformation for the duration of the simulation of wild type Btk linker-kinase (Fig 3A and 3E) but transitioned from the active “C-in” to inactive “C-out” conformation at early time points in the simulation of inactive Btk (W395A) linker-kinase (Fig 3B–3D and 3F). The αC-helix transition captured in the Btk (W395A) linker-kinase simulation led to loss of the K430/E445 salt bridge and concomitant changes in the electrostatic contacts between R544 and pY551 (Fig 3F). In contrast, the distance between the K430/E445 and R544/pY551 side chains remained constant throughout the wild type Btk linker-kinase domain simulations (Fig 3E). Consistent with conformational changes localized to the regulatory regions in the N-lobe, the RMSD of total backbone atoms of Btk linker-kinase domain and Btk (W395A) linker-kinase showed only small changes throughout the simulation time (Fig 3A–3D).

The N-terminal region of the activation segment (residues 540–547) also behaved differently in simulations of wild type Btk linker-kinase versus (W395A) linker-kinase domain (Fig 3A–3D). The activation segment sampled conformations near the active state during the simulation of the Btk linker-kinase domain (Fig 3A). In contrast, the activation segment in Btk (W395A) linker-kinase drifted to a greater extent from the starting active conformation as indicated by the greater RMSD from its starting active conformation (Fig 3B–3D). The DFG motif at the N-terminal end of the activation segment retained its active conformation during the simulation of the Btk linker-kinase domain but reverted to the conformation seen in the crystal structure of inactive Btk in simulations of Btk (W395A) linker-kinase (Fig 3F). The activation loop as a whole, however, did not transition into the inactive conformation seen in the crystal structure of inactive Btk (3GEN) likely due to the length of simulation time and the fact that the activation loop tyrosine, Y551, is phosphorylated in the simulations (Fig 3F). Overall, the conformational changes observed in the simulations of Btk (W395A) linker-kinase domains suggest that the mutant kinase is not stable in the active conformation, consistent with the experimental observation that the side-chain of W395 plays a critical role in maintaining the active conformation of the Btk kinase domain.

### W395A changes the dominant motions within the Btk kinase domain

**Fig 4:**
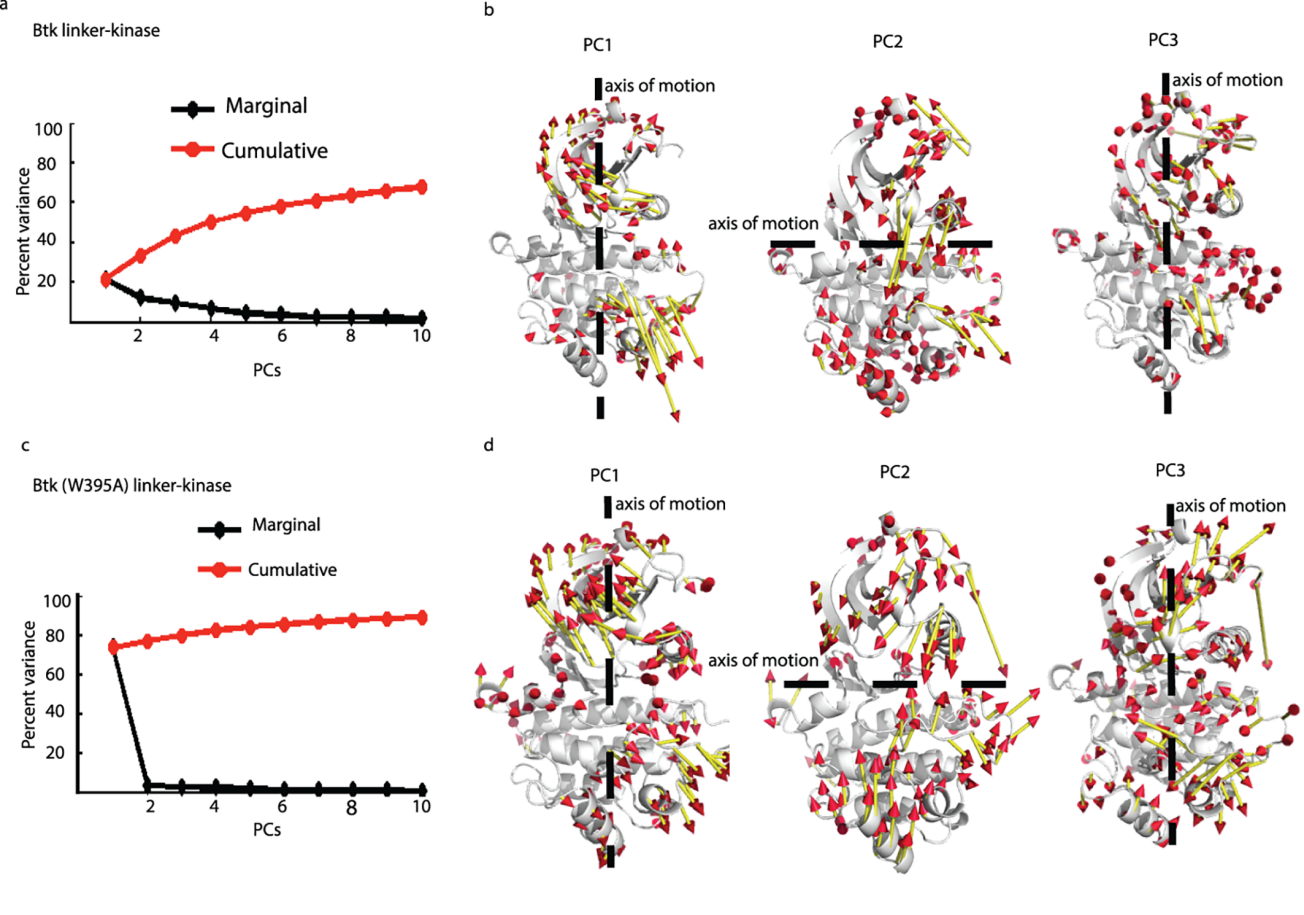
Principal Component Analysis. (a,c) Percent variance captured by the first 10 PCs in Btk linker-kinase (a) and Btk (W395A) linker-kinase (c) domains. The red line is the cumulative variance captured by the PCs and the black line is the percentage of the variance captured by each individual PC. (b,d) Direction of motions in PC1, PC2 and PC3 for Btk linker-kinase (b) and Btk (W395A) linker-kinase (d) domain. Dotted lines show the axis of motion, the length of the vectors show the relative magnitudes and the arrowheads indicate the direction of motion.

Principal Component (PC) Analysis (Fig 4, see Figs B and C in S1 Text) reveals the important motions in the system that might otherwise be obscured by the small fluctuations within a trajectory [21]. The first few PCs can capture a large fraction of the variance in the data and thus represent the dominant motions. We calculated the root mean-square inner product (RMSIP) between the first 10 PCs from each of the trajectories for Btk linker-kinase and of trajectories for Btk (W395A) linker-kinase domain to determine how well the PC subspace overlaps between the different replicates. Large RMSIP values were seen between the subspaces covered by first 10 PCs from each of the replicates for both Btk linker-kinase and Btk (W395A) linker-kinase domain simulations (Btk linker-kinase domain, rep1 vs rep2: 0.73, rep1 vs rep3: 0.7, rep2 vs rep3: 0.68. Btk (W395A) linker-kinase domain, rep1 vs rep2: 0.68, rep1 vs rep3: 0.66, rep2 vs rep3: 0.64), indicating that there is high similarity between the sets of PCs derived from each of the individual replicates. The first three PCs in Btk linker-kinase and Btk (W395A) linker-kinase domain simulations captured 43.37% and 79.8%, respectively, of the total variance observed in the simulation data (Fig 4A and 4C). The dominant motion within Btk linker-kinase domain, as captured by the first three dominant principal components include an opening-closing motion of the N- and C-lobes around a hinge (Fig 4B, PC2). A similar ‘breathing’ motion was described for other active kinases [4, 22, 23], suggesting this is a shared feature of catalytically competent kinases and may be responsible for the greater amide NH accessibility observed for the active protein with HD exchange methods. Indeed, this ‘breathing’ motion is considered important for the structural rearrangement of the αC-helix and activation segment to assemble the active site and is considered necessary for the release of ADP after the reaction is complete [4, 24]. Overlap between PCs derived from the three replicates of each Btk linker-kinase shows that high overlap exists between PCs capturing the ‘breathing’ motion (see Fig D in S1 Text). In contrast, the dominant mode in the Btk (W395A) linker-kinase domain simulation, captured almost entirely by its PC1, shows a combination of twisting and translation motion of the N- and C-lobes (Fig 4D) quite similar to that seen in simulations of other inactive kinases [22, 23]. Overall, differences in PCs are consistent with the conclusion that W395A alters the global motions of the Btk kinase domain.

PCA was also performed by combining the three simulation replicates of Btk linker-kinase together with the Btk (W395A) linker-kinase domain by aligning to the same reference structure to compare the overlaps between the simulation trajectories along the PC1-PC2-PC3 of this collective subspace (see Fig D in S1 Text). It is clear from the distribution of the trajectories that the mutant Btk (W395A) linker-kinase domain (see Fig D in S1 Text) samples a sub-space that is mostly different from that sampled by the wild-type Btk linker-kinase domain (see Fig D in S1 Text) but does include sampling of a part of the sub-space similar to the wild-type Btk linker-kinase.

### Community analysis reveals dynamic coupling throughout the kinase domain is lost on mutation of W395 to alanine

**Fig 5:**
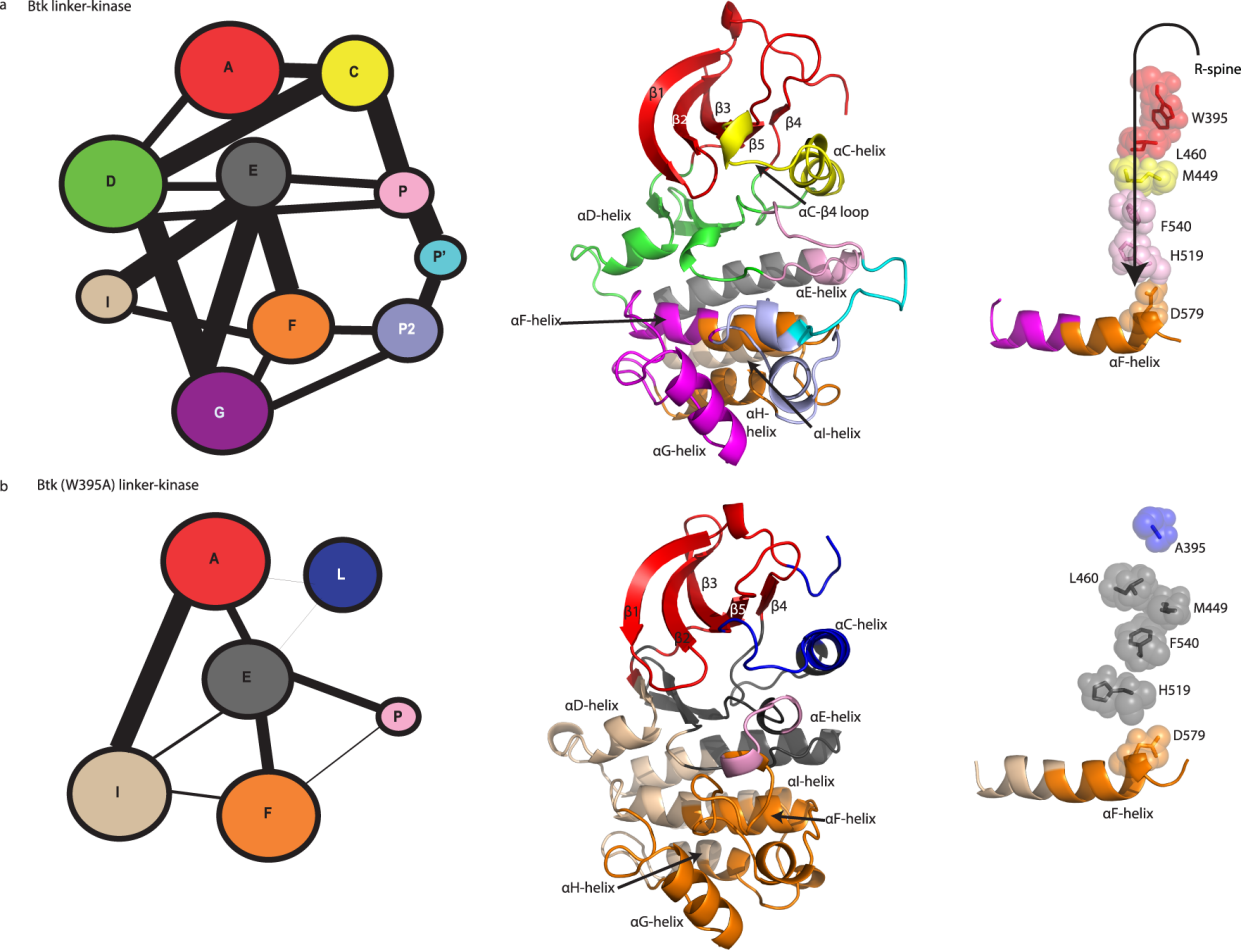
Community analysis. The community network in Btk linker-kinase (a) and Btk (W395A) linker-kinase (b) domains. (a,b *left panel*) The area of the circle indicates the number of residues within each community and the weight of the lines connecting communities is proportional to the extent of correlation between communities. (*middle panel*) Communities of residues mapped onto the Btk linker-kinase domain structure for Btk linker-kinase (a) and Btk (W395) linker-kinase (b). (*right panel*) R-spine communities for the Btk linker-kinase simulation (a) and the Btk (W395A) linker-kinase simulation (b).

PCA provided a broad picture of the relative motions of the N- and C-lobes for the wild type structure and for the mutant W395A. To identify dynamic sub-segments within each lobe of the Btk kinase domain, we next examined the correlation of motions of residue pairs within the kinase domain of Btk linker-kinase and the (W395A) linker-kinase mutant. C^α^ coordinates were used for this analysis, as coarse-grained models have proven valuable in capturing the intrinsic dynamics of proteins [25, 26]. The cross-correlation coefficients for Btk linker-kinase and Btk (W395A) linker-kinase were used to build dynamic cross-correlation maps to examine the differences in the correlated motions that remain within 10 Å for 75% of the simulation time (see Fig E in S1 Text). The 10 Å cutoff was chosen as it has been found to be the optimal cutoff for modeling interactions in coarse grained models as well as for comparison of our results with community analysis results of other active kinases such as PKA [27]. The cross-correlations were then used to build a correlated network of residues; represented as a set of connected circles (communities) with the weight of the lines connecting each community proportional to the degree of their correlation (Fig 5, see Fig F in S1 Text). The residues of wild type Btk linker-kinase and Btk (W395A) linker-kinase clustered into distinct communities (Fig 5A and 5B), reflecting difference in the dynamic sub-segments of the active versus inactive kinase domain. Detailed summaries of the communities are provided in Fig G in S1 Text. Where possible, we have labeled the communities (Com) in Btk linker-kinase in a manner similar to those described by McClendon and co-workers in their community analysis of PKA [27].

Community clustering based on cross-correlations identified two major communities, ComA, and ComC, in the Btk linker-kinase domain N-lobe (Fig 5A). All of the linker residues including W395 are part of ComA, which also contains beta strands β1, β2, β3, β4 and β5, regulatory spine residue L460 and the Gly-rich loop. ComC includes the important αC-helix and the R-spine residue M449. Separate from the αC-helix, the αC- β4 loop segregates with ComD, which extends into the C-lobe and includes the hinge region of the kinase domain. The catalytic loop HRD motif including the R-spine residue H519 and the N-terminus of the activation segment, which includes the regulatory spine residue F540 (part of the DFG motif) are clustered in ComP. The grouping of disparate regions of the kinase domain into communities A, C, D and P points to dynamic coordination in the active Btk linker-kinase domain. Indeed, the assembled R-spine residues reside in four different communities demonstrating the correlation of this structure to different regions in the active kinase domain.

It is immediately evident that there are fewer communities within Btk (W395A) linker-kinase domain and the correlations between these communities are weaker compared to those of the wild type Btk linker-kinase protein (Fig 5B). This result suggests that a mutation that inactivates the kinase domain may do so by reducing as well as dampening correlated motions within the protein. The integrity of ComC was lost in the Btk (W395A) linker-kinase domain, where the αC-helix itself is divided into two dynamically distinct communities ComL and ComE. The αC- β4 loop in the inactive Btk (W395A) linker-kinase is in ComE, which is separated from the hinge region in ComI. The R-spine residues within Btk (W395A) linker-kinase are dynamically isolated in ComE, which is only weakly linked to other structurally significant regions of the kinase domain (Fig 5B). Comparing community analysis of the wild type and mutant Btk kinases indicates that loss of the single W395 side-chain results in a breakdown of the “signal integration” function [27] [28] of the αC-helix across the two lobes of the kinase with R-spine assembly and dynamics being adversely affected.

### Specific residues mediate allosteric communication

**Fig 6:**
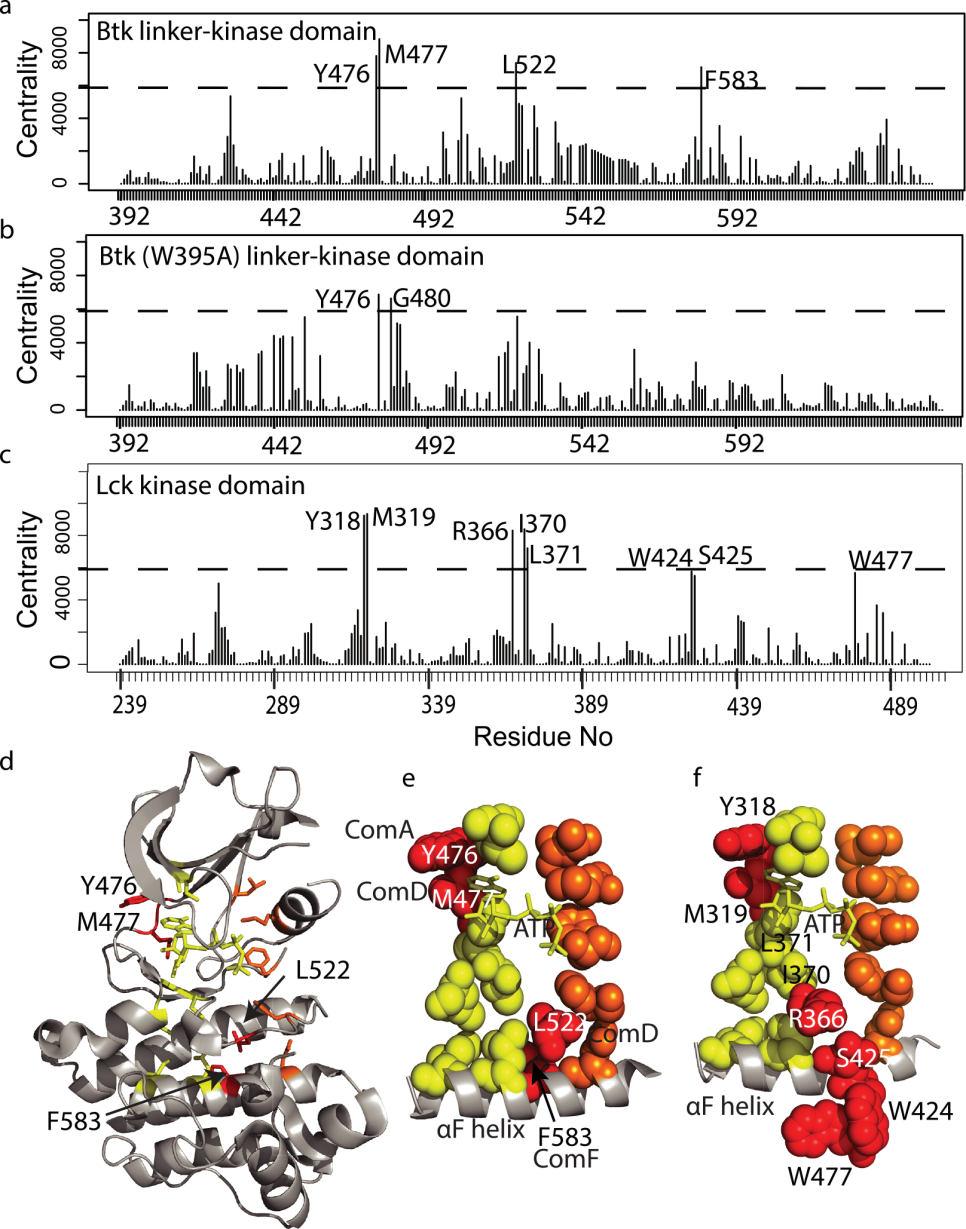
Node-betweenness centrality index values reveals residues that bridge the R- and C-spines. (a-c) Node-betweenness centrality index plot for Btk linker-kinase (a), Btk (W395A) linker-kinase domains (b) and Lck kinase domain (c). The threshold (dotted line) was set for Btk linker-kinase (a), such that 98.5% of the centrality index values are below the threshold value. The same threshold is used for Btk (W395A) linker kinase in (b) and Lck in (c). In (a) and (c) the centrality value for one residue, A428 in Btk and A271 in Lck, nearly reaches the threshold but was not included in our analysis since this residue is part of the previously defined C-spine in both kinases. (d) High centrality residues from (a) are mapped onto the structure of active Btk (3K54), labeled, and colored red. C-spine residues are yellow and R-spine residues are orange as in Fig 1B. (e) Spheres define the residues of the C-spine (yellow), R-spine (orange) and the newly identified bridging residues (red). The αF-helix is shown and ATP within the C-spine is depicted in stick form. The communities (Fig 5) of each of the four bridging residues are indicated. (f) High centrality residues in Lck (shown in (c)) are mapped onto the structure of the active Lck kinase domain (3LCK), labeled and colored red. As in (e) Lck C-spine residues are yellow and R-spine residues are orange, the αF-helix is shown and ATP is shown in stick form. I370 and L371 are high centrality residues in Lck (see (c)) and are part of the previously defined C-spine and therefore colored yellow.

To identify specific residues responsible for the allosteric communication between W395 and the rest of the kinase domain, we next computed the node-betweenness centrality index for each residue within both the wild type Btk linker-kinase (Fig 6A) and the Btk (W395A) linker-kinase (Fig 6B). Centrality index is a measure of the number of shortest paths that pass through a node, which is a direct measure of the contribution of the node to the total communication flow in the system [29, 30]. High centrality values correlate with the importance of these nodes in the transmission of information through the network. This analysis therefore identifies residues that act as hubs in the allosteric communication pathway transmitting the activating effect of W395 throughout the Btk kinase domain.

Four residues, Y476, M477, L522 and F583 within the active Btk linker-kinase domain exhibit high centrality while in Btk (W395A) linker-kinase domain only two residues, Y476 and G480, are above the same threshold centrality value (Fig 6A and 6B). In wild type active Btk the hub residues are located in two regions, Y476 and M477 appear to complete the C-spine and wrap around ATP pocket in the N-lobe (Fig 6D and 6E) and L522 and F583 are situated between the base of the C- and R-spines in the C-lobe (Fig 6D and 6E). The location of L522 and F583, in particular, suggest a bridging role where these residues might function to communicate R-spine assembly to the C-spine and the ATP bound active site. Along these lines it is interesting to note that while M477 and L522 are 14Å apart and are located in different lobes of the kinase domain, they both belong to ComD (Fig 5A) suggesting a central, and correlated, role in the allosteric dynamics of kinase activation.

The now well recognized C- and R-spines are a shared feature of the kinase family and so we wished to assess whether similar bridging residues would be detected in another active kinase domain. Simulation data acquired previously for the Src family tyrosine kinase, Lck [17] were used to compute the centrality index values for each residue of the active Lck kinase domain. A set of Lck residues with high centrality values emerged (Fig 6C) that are quite similar to those found for Btk (Fig 6A). The high centrality Lck residues include Y318 and M319 that correspond exactly to Y476 and M477 in Btk (Fig 6F). As well, Lck residues R366 and S425 are located between the base of the C- and R-spines in a manner similar to L522 and F583 in Btk. Two additional Lck residues in the C-lobe, W424 and W477, reach the high centrality threshold and the C-spine residues I370 and L371 exhibit high centrality in Lck. The fact that a similar arrangement of high centrality residues are identified in two different active kinases and that a subset of these residues bridge the R- and C-spines provides further support for a mechanistic model for activation whereby regulatory spine assembly may be dynamically communicated to the catalytic apparatus of the active site.

## Discussion

Tryptophan 395 is a positive regulator of Btk kinase activity [11]. In contrast to the negative regulatory role of the same tryptophan in the Src family kinases [14, 31], mutation of this single residue abolishes the catalytic activity of the 70 KDa full-length Btk kinase. Our experimental and computational findings provide an explanation for the positive allostery observed for the Tec family and suggest that a particular conformation of the W395 side-chain promotes long range correlated dynamic motions throughout the kinase domain that are essential for catalytic activity.

MD simulations indicate that the Btk αC-helix samples the ‘C-in’ state for the majority of the trajectories when W395 adopts the rotameric conformation observed in the crystal structures of active Btk. In contrast, the absence of the W395 side-chain, achieved either by a rotameric shift or by mutation to alanine, favors the ‘C-out’ state. The conformational preferences of the αC-helix are linked to the overall motions of the N- and C-lobes of the kinase domain, as well as to the degree of correlation between residues throughout the kinase domain. Based on the computational work presented here and by others [27, 32, 33], and previous analyses of active and inactive kinases by NMR spectroscopy [34–36], it is becoming clear that the active state of a kinase domain requires a specific highly dynamic, interconnected structure. The inactive kinase exhibits substantially less cohesion, with fewer correlated motions throughout the kinase domain when motions are compared with those of the active form. Moreover, the hydrogen/deuterium exchange data indicate that specific regions of the wild type Btk linker-kinase domain sample more open conformations compared to the Btk (W395A) linker-kinase mutant, consistent with the view that inactivating mutations suppress the dynamics required for catalytic activity.

While it may be tempting to explain allostery in terms of a simple pathway leading from the W395 side-chain to the kinase active site, our findings, as well as the work of others [27, 32, 37], argue against a simple linear allosteric pathway. In a recent review focusing on dynamics and allostery within the protein kinases [38], Kornev and Taylor drew an elegant analogy between the kinase domain and the violin, invoking the vibrations responsible for the tone and pitch emanating from a violin as a way to think about the role of molecular dynamics in controlling the catalytic activity of the enzyme [38]. Small, localized changes on the violin, such as placing a finger on a string, can have a dramatic effect on the resulting pitch by altering the vibrations of the entire instrument. Amino acid mutations are similarly likely to affect protein function by altering the long range correlated motions throughout a structure, and changes at particular positions may critically affect function. Indeed, our data suggest that the inactivating W395A mutation substantially dampens the dynamics of the Btk kinase domain in much the same way that muting restricts vibrations throughout a violin by reducing the volume of the notes.

Community analysis [39] provides insight into changes in correlated motions that result from ligand binding and/or amino acid mutation [27, 32, 33, 40, 41]. The set of communities identified within Btk are similar to those defined previously for PKA [27]. In both, the communities contain residues from distant regions of the primary structure and connections between certain communities are strong, indicating that correlations within an active kinase are spread throughout the kinase domain. Analyses of inactive Btk mutants reveal fewer communities that are less coherent indicating that the correlated motions typical of an active kinase are absent in the inactive state.

We have extended our analysis separately to the Btk linker-kinase and the Btk (W395A) linker-kinase to include computation of the residue specific node-betweenness centrality index [42]. A higher centrality indicates that a residue serves as a node or hub, playing a greater role in information flow in the network. In the active Btk linker-kinase and Lck kinase domains, specific residues across the primary structure are characterized by high centrality values suggesting their involvement in the flow and transmission of dynamical information in the active state. The similarity in tertiary structural arrangement of these residues in both kinases is compelling and two of the high centrality residues in each active kinase are perfectly positioned between the two well-characterized C- and R-spines suggesting a bridging role. In terms of kinase activation, the dynamical consequences of the R-spine assembly (triggered by activation loop phosphorylation and a shift to the ‘C-in’ state) may be transmitted to the catalytic machinery of the kinase domain via these bridging residues, thereby integrating assembly and catalysis within the active kinase. Sequence conservation within each kinase family underscores the importance of the bridging residues and for Btk it is noteworthy that all four high centrality residues are sites of XLA mutations. Whether these XLA mutations specifically disrupt communication between the R- and C-spines to prevent kinase activation or simply alter the overall fold and stability of the Btk kinase domain remains to be determined. As was the case with the R- and C-spines, the true importance of the high centrality residues in each kinase must be tested experimentally.

It is generally challenging to clearly delineate the structural consequences of deleterious point mutations. Yet understanding precisely how specific mutations disable enzymes can provide the information necessary to pursue new strategies toward modulating protein functions for therapeutic applications. Here, by combining experiment with pertinent simulations we establish the detailed requirements for a particular rotameric conformation of a native tryptophan for activity and relate this small region of the structure to the dynamics of the entire catalytic unit. We have seen how a single point mutation can destroy a protein’s activity by modifying its dynamics and allostery. The results found here are consistent with the growing realization of the importance of conservation for functional dynamics [1, 38, 43, 44], and the present case provides strong support for this view.

## Methods

### Constructs, protein expression and activity assays

Baculoviral and bacterial constructs, protein expression and purification conditions are described elsewhere [11, 45]. The W395A mutation was introduced using site-directed mutagenesis (Stratagene) and verified by sequencing. In vitro kinase assays were performed as previously described [35].

### HDXMS

Duplicate deuterium labeling experiments were initiated with an 18-fold dilution of an aliquot (63 pmoles) of Btk linker-kinase or Btk (W395A) linker-kinase into a buffer containing 99.9% D_2_O, 20 mM Tris, 150mM NaCl, 10% glycerol, pD 8.01. The labeling reaction was quenched by addition of quench buffer (150 mM potassium phosphate (pH 2.47)) at 10 secs, 1 min, 10 mins, 1 hour, and 4 hours. Quenched samples were immediately injected into a Waters nanoACQUITY with HDX technology [46] for online pepsin digestion and ultra-performance liquid chromatography (UPLC) separation of the resulting peptic peptides, and analyzed as reported previously [17]. All mass spectra were acquired with a WATERS SYNAPT G2si HDMS mass spectrometer. The data were analyzed with DynamX 3.0 software. Relative deuterium amounts for peptides covering 97.9% of the protein backbone were calculated by subtracting the average mass of the undeuterated control sample from that of the deuterium-labeled sample for isotopic distributions corresponding to the +1, +2, +3, or +4 charge state of each peptide. Data were not corrected for back exchange and are reported as relative values [15]. Differences larger than 0.7 Da are considered obvious, according to the statistical criteria for relative HDXMS measurements previously described [47].

### Structure preparation

The coordinates of Btk linker-kinase (PDB ID: 3K54, amino acids: 392–659) were obtained from the RCSB PDB databank [20]. The coordinates of the bound inhibitor were deleted from the PDB file. The regions missing from the electron density maps of 3K54 were modeled with the Loop Model module in MODELLER as follows [17]: amino acids 435–441, which include the β3- αC loop and the N-terminus of the αC-helix, were modeled using Csk (PDB ID: 1K9A, chain B) [48] and Lck (PDB ID:3LCK) [49] as templates; amino acids 542–558 which form the activation loop are modeled based on Btk (PDB ID:1K2P) [50] since 1K2P contains the activation loop resolved in the open conformation. Finally, none of the available crystal structures of Btk contain the DFG motif in the active conformation and so we used the structure of the active Lck kinase domain (PDB ID:3LCK) to model the active DFG-in conformation into Btk 3K54. The mutate_model module in Modeller was used to mutate W395 to alanine in Btk linker-kinase domain model to derive Btk (W395A) linker-kinase domain. Phospho-Tyrosine patch TP2 was used to introduce phosphorylation of Y551 in both models.

### Simulation setup

The NAMD 2.8 [51] program with CHARMM27 [52] force field was used to initiate all-atom MD simulations of Btk linker-kinase and Btk (W395A) linker-kinase. The proteins were solvated in a periodic water box with 15 Å buffering distance between protein surface and the box, using the TIP3P explicit water model. 150 mM concentration of ions (Na+ and Cl-) was added to charge neutralize the system. The systems were equilibrated and simulated in the NPT (Normal Pressure Temperature) ensemble at 310 K and 1 atm, using Particle-Mesh Ewald for long-range electrostatics. The cutoff used for the van der Waals and short-range electrostatics calculations was 12 Å and hydrogen bonds were kept rigid using the ShakeH algorithm. The timestep used was 2 fs.

The prepared simulation systems were minimized according to the following steps: (a) 20 picoseconds (ps) minimization of the entire system followed by 50 ps of equilibration by holding the protein rigid, allowing only water molecules and Na+ and Cl- ions to move. (b) The modeled loops which included the Gly-rich loop and the activation segment as well as the β3-αC-helix loop were subjected to a very short minimization of 2 ps to remove any steric clashes. (c) The entire system was minimized, gradually releasing harmonic constraints on all protein heavy-atoms. The temperature of the system was then gradually raised from 200 K to 310 K with harmonic constraints on all protein heavy-atoms, in 5 K increments over a total of 90 ps. Subsequently, the harmonic constraints were gradually released and the system was equilibrated for a total time of approximately 1 ns.

The production MD was run for 200 ns each. The simulations were run in triplicates.

### Simulation trajectory analysis

VMD [53] was used to visualize the simulations trajectory and calculate Root Mean Square Deviation (RMSD) as well as salt-bridge and hydrogen-bonding distances. For RMSD calculations, superposition is based on the C-lobe (N479-S659) using the energy-minimized structure as a reference. MATLAB (The Mathworks, Inc.) was used to plot RMSD and distances obtained from VMD. Figures were generated with PyMOL[54].

### Principal Component Analysis

C^α^ coordinates of Btk linker-kinase and Btk (W395A) linker-kinase domain from 200ns MD trajectory are the input for PCA. PC analysis was carried out as described before [21]. MATLAB was used for the above calculations. Directions of motion in PC1-PC3 were mapped onto the structure using the modevectors script in pymol [54]. To compare the overlap between the simulation trajectories, the PC scores were projected along the PC1-PC2-PC3 subspace using MATLAB, after combining all the simulation trajectories and aligning them to the same reference structure. Root Mean Square Inner Products (RMSIP) between the first 10 PCs in each of the replicate of Btk linker-kinase and Btk (W395A) linker-kinase domains were calculated using the Bio3d [55] in R [56] as described before [21].

### Cross-correlation analysis

Cross-correlation coefficients indicate whether points in a system move in the same or opposite direction and are correlated or move in orthogonal directions, in which case the method does not pick up correlations. The C^α^ atoms of each protein were aligned to the first frame of the trajectory. The dot products of the displacements Δr of C^α^ atoms are used in the correlation coefficients (C_ij_) as follows:

$$C_{\text{i},\text{j}}=\frac{<\Delta r_{\text{i}}•\Delta r_{\text{j}}>}{{\left({\left|\Delta r_{\text{i}}\right|}^{2}{\left|\Delta r_{\text{j}}\right|}^{2}\right)}^{1/2}}$$

where Δr_i_ denotes the displacement of residue i from the mean. The pairwise cross-correlation coefficients between pairs of residues form the elements of the cross-correlation matrix. The values of the cross-correlation coefficients range between -1 and 1, with -1 denoting negative-correlation, +1 denoting positive-correlation and 0 denoting no-correlation. The matrix is depicted as a dynamic cross-correlation map (DCCM).

### Community clustering and node-betweenness analysis

The Girvan-Newman clustering algorithm [39] was used to identify communities of residues from the set of correlated residues obtained above. Cross correlation coefficients (C_ij_) whose absolute values are below the set cutoff of 0.5 were ignored in building the unfiltered DCCM. A proximity/contact map filter was applied for building the correlation network of residues for those within 10 Å of one another for at least 75% of simulation time [27]. A network is the interconnected set of amino acid residues or nodes. Communities are identified using the edge “betweenness” approach, which is defined as the number of shortest paths between a pair of nodes (amino acid residues). The size of the community is the number of amino acids that have a high degree of correlated motion (depicted by size of circle), while the thickness of the edges/links connecting the communities denotes the extent of correlation.

The middle 100 ns (from 50ns-150ns of total of 200ns) of simulation data was used to calculate the centrality in both Btk linker and Btk (W395A) linker-kinase domains. Our rationale in using this time-window is that we wished to pick up the communication hubs (amino acids), which mediate the conformational change from the active to inactive state as the most prominent conformational change in RMSD is observed in the Btk (W395A) linker-kinase domain simulation during this time interval. The corresponding simulation time-scale (50ns-150ns) for Btk linker-kinase domain was also analyzed. We decided to not include simulation trajectory beyond 150 ns as it introduced noise in the node-betweenness calculations. Node-betweenness is an approach complementary to edge-betweenness, wherein the number of unique shortest paths passing through a node are counted. The cross-correlation analysis, community clustering and node-betweenness calculations, were carried out using the dccm and cna functions in Bio3d package [55] in R [56].

## Supporting Information

S1 Text Supporting figures. (DOCX)
