# Supplementary material for: Dynamic Allostery Mediated by a Conserved Tryptophan in the Tec Family Kinases
Source: PLoS Comput Biol. 2016 Mar 24;12(3):e1004826. doi: 10.1371/journal.pcbi.1004826 (PMC4807093; doi:10.1371/journal.pcbi.1004826)
Supplement: S1 Text — (DOCX) [file pcbi.1004826.s001.docx]

S1 Text

**
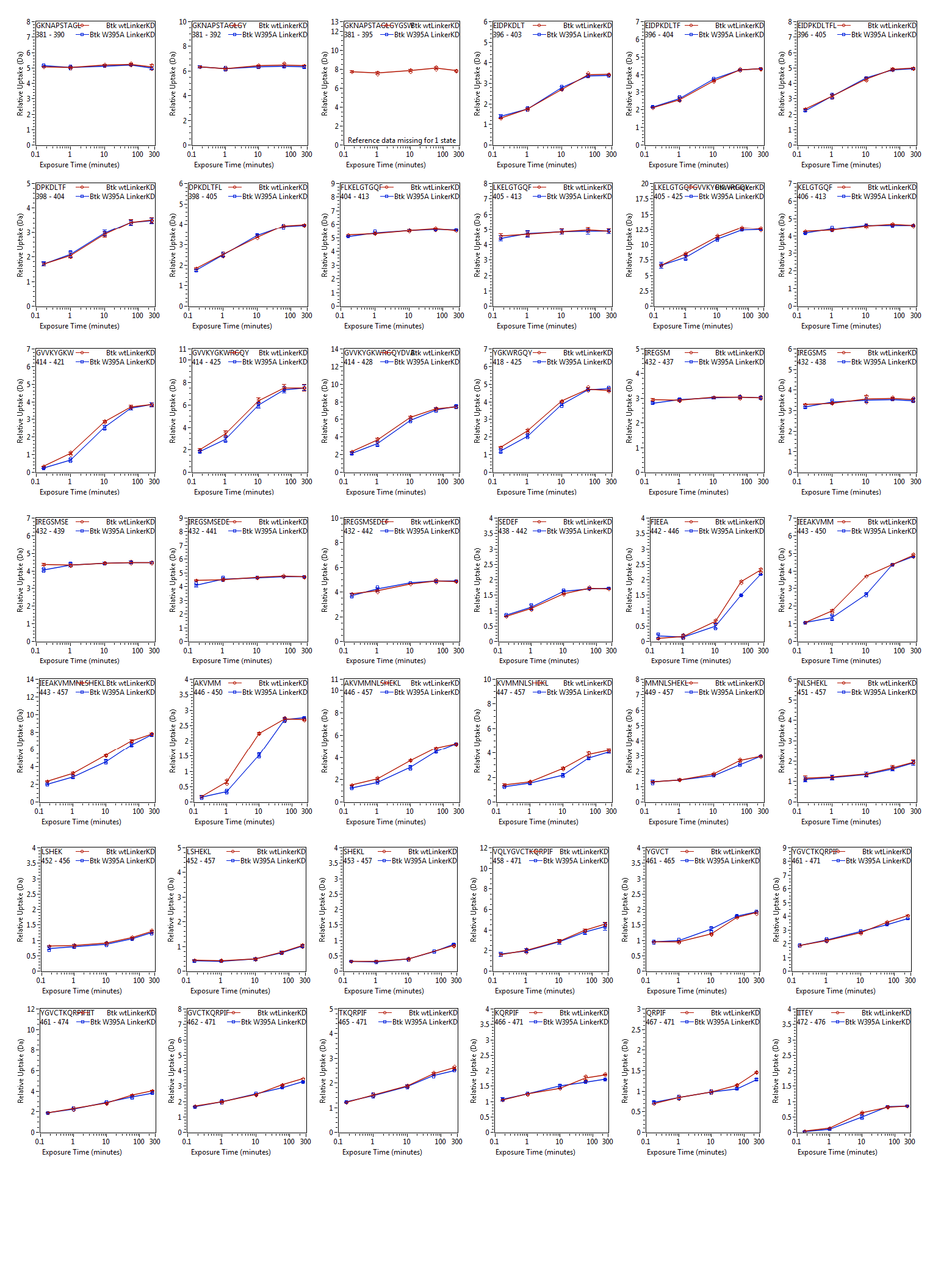
**

**
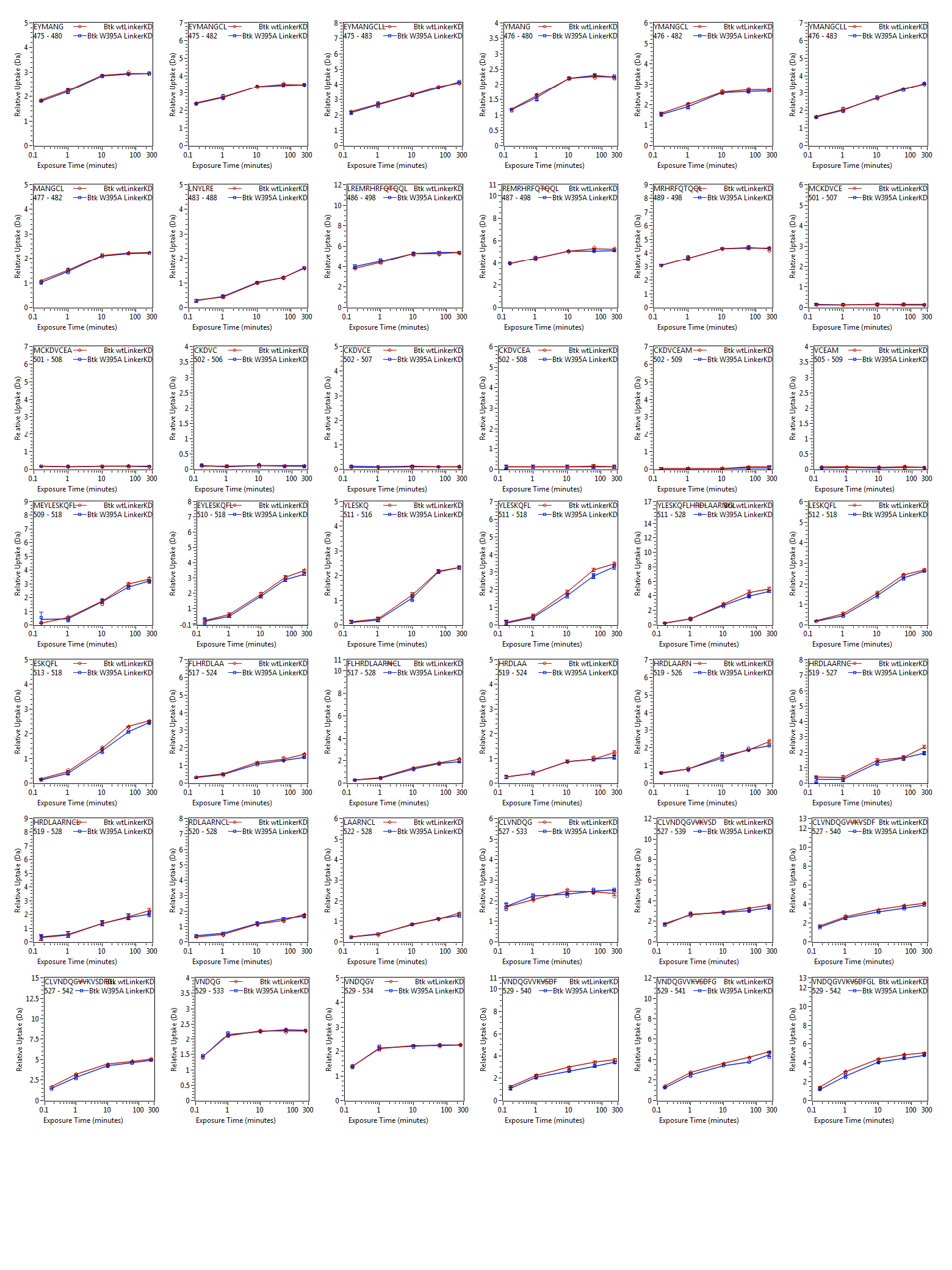
**

**
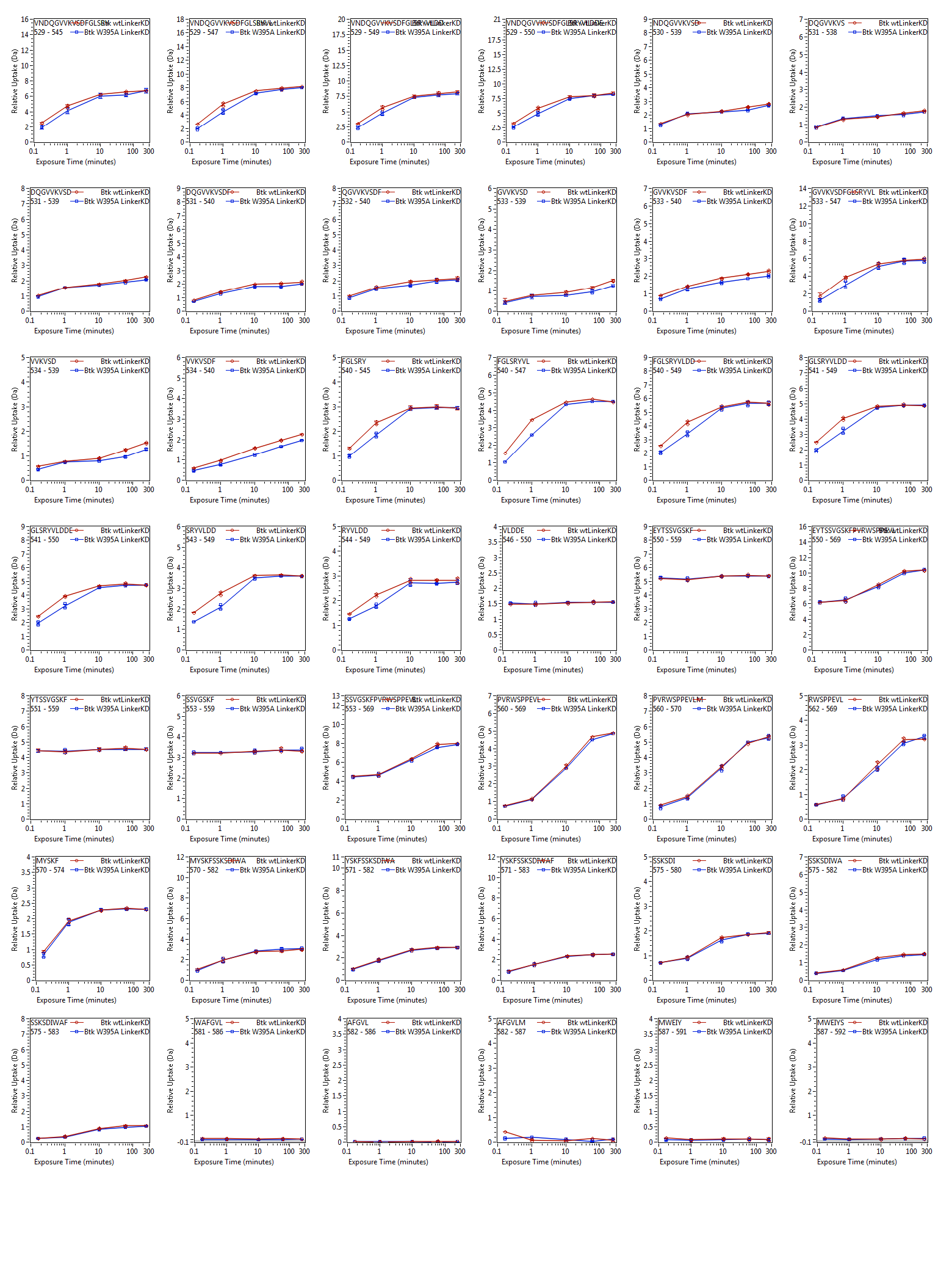
**

**
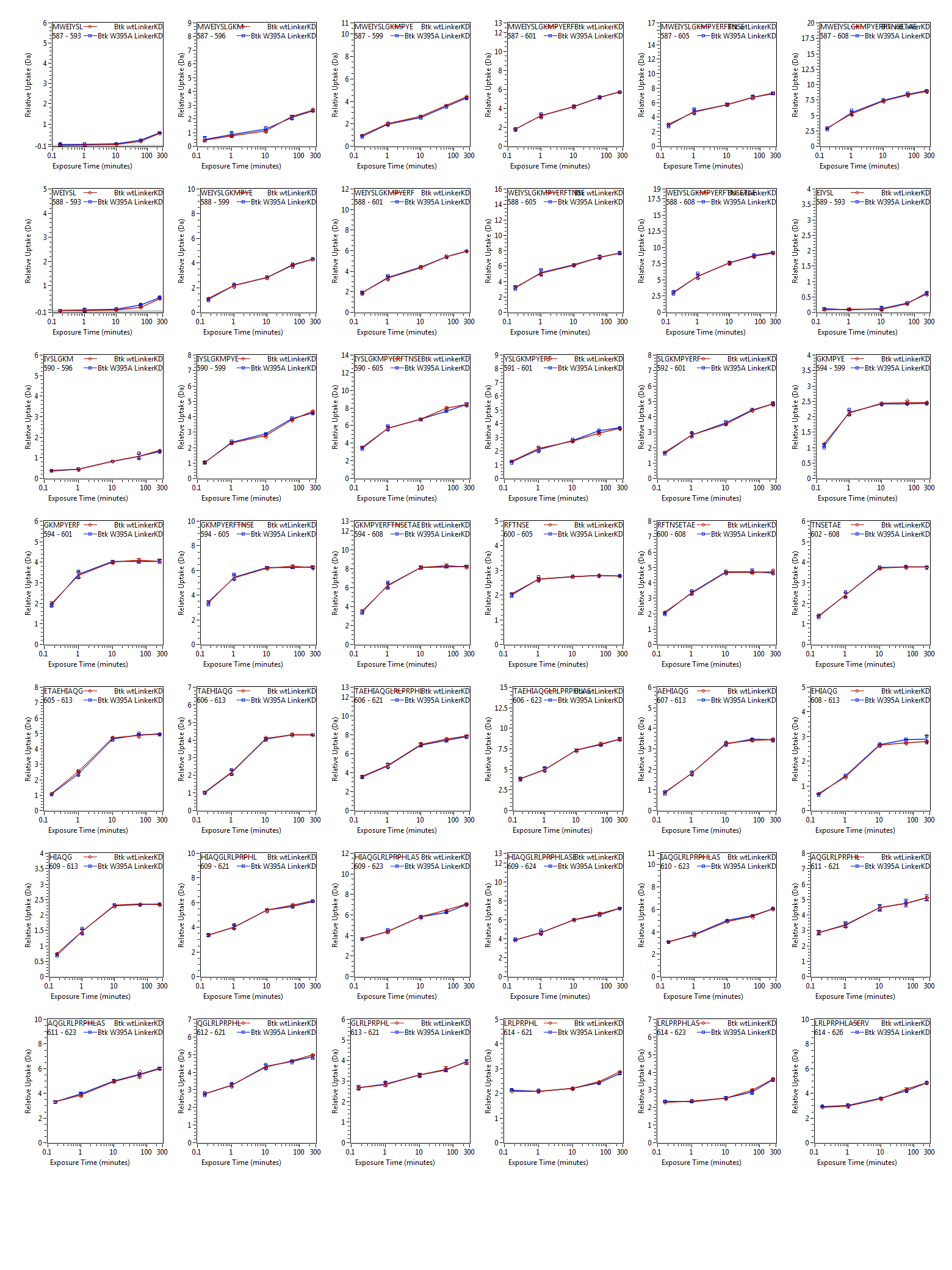
**

**
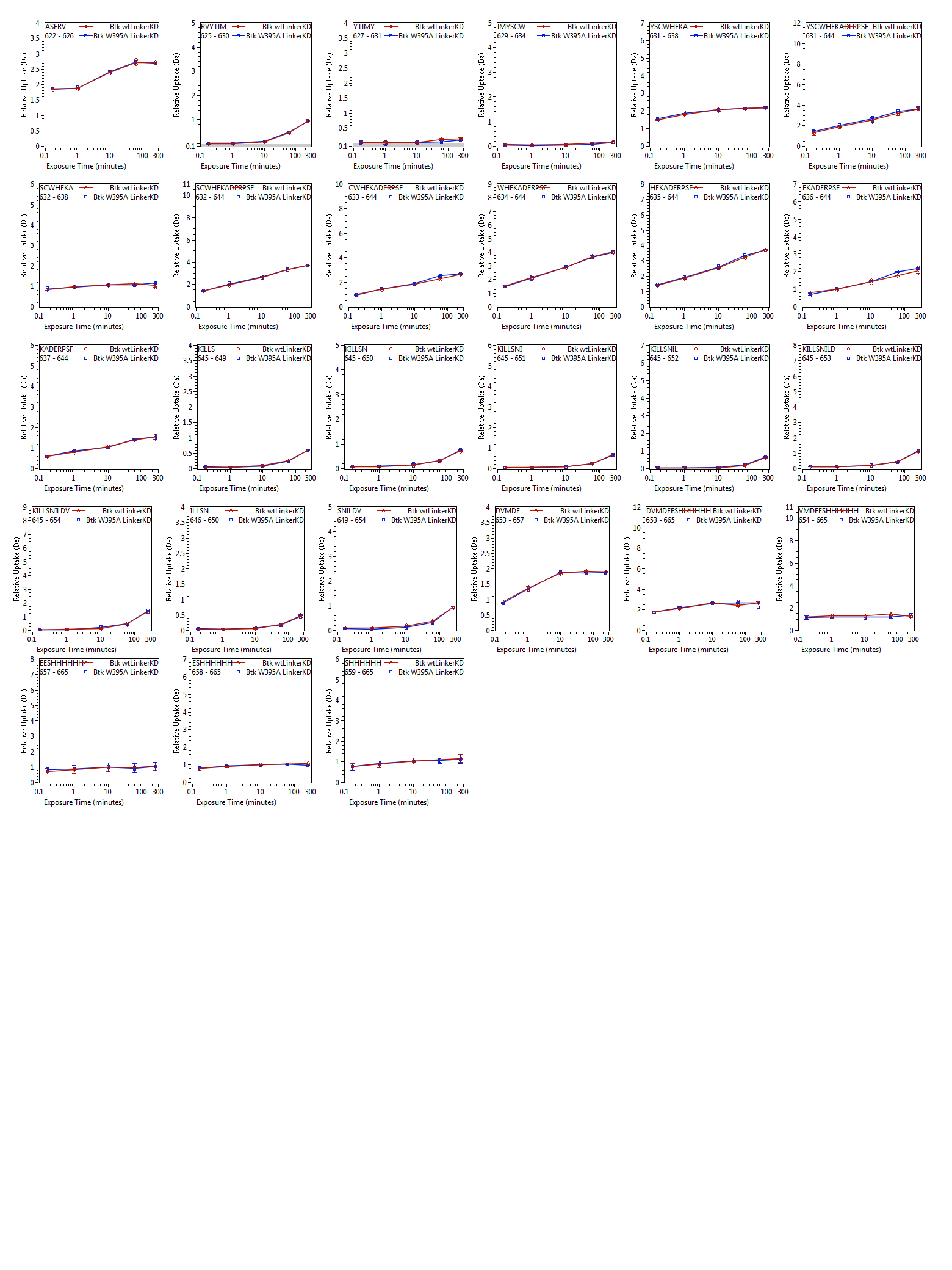
**

**Fig A Complete HDX data for Btk linker-kinase and Btk (W395A) linker-kinase**

**
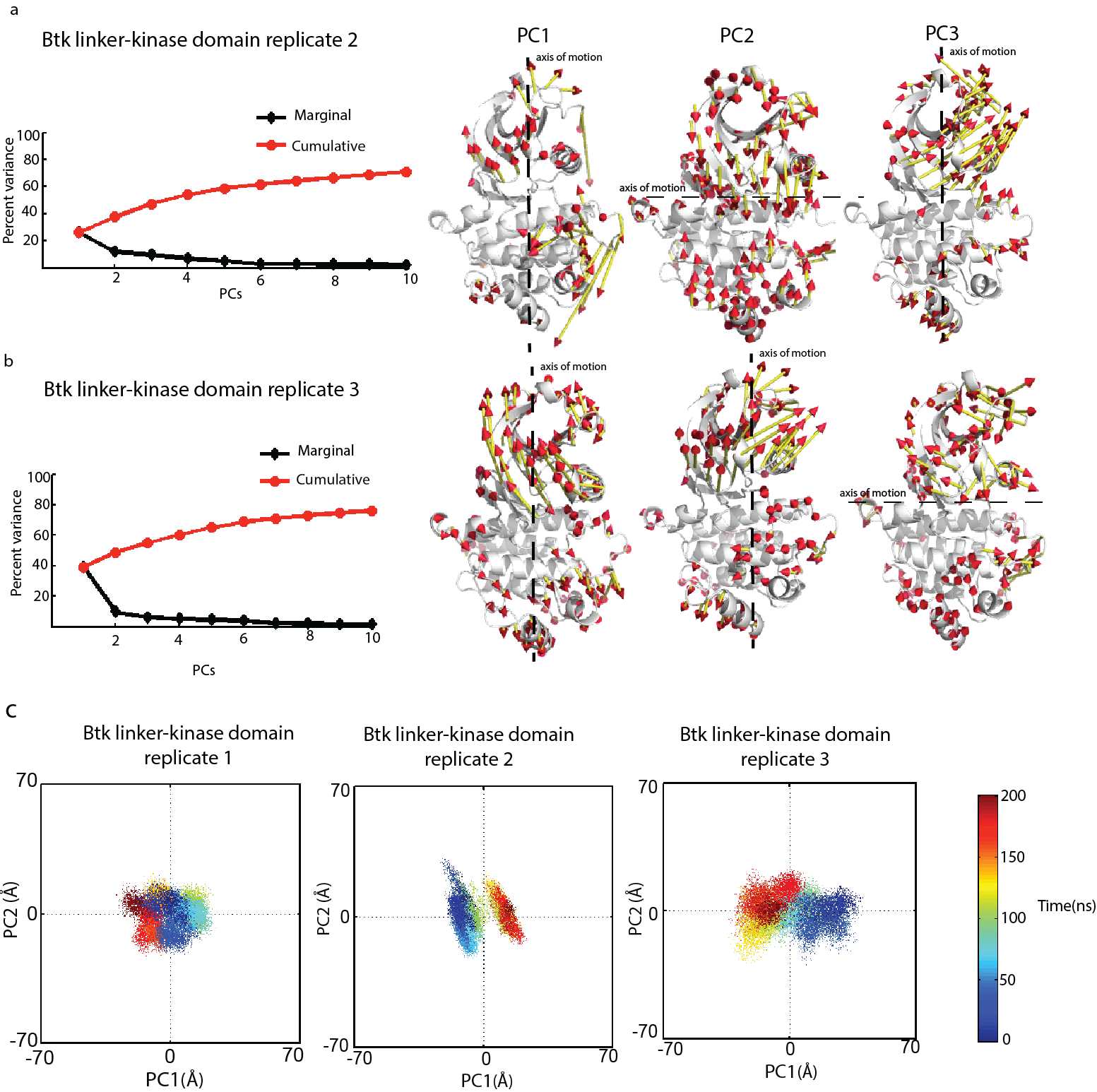
**

**Fig B PCA : Btk linker-kinase domain** Percentage of variance captured by the first 10 Principal Components (PC) in replicate 2 (a) and replicate 3 (b) for the Btk linker-kinase domain. The red line is the cumulative variance captured by the PCs and the black line is the percentage of the variance captured by each individual PC. Directions of motion for the first three PCs for the second (a) and third replicate (b) of Btk linker-kinase are shown alongside the percent variance plot. The length of the vectors show the relative magnitudes and the arrowheads indicate the direction of motion. (c) The PC scatter plots showing the projections of the conformations along PC1 and PC2 space, for the first, second and third replicates of Btk linker-kinase domain are shown. The color bar indicates simulation timescale.

***
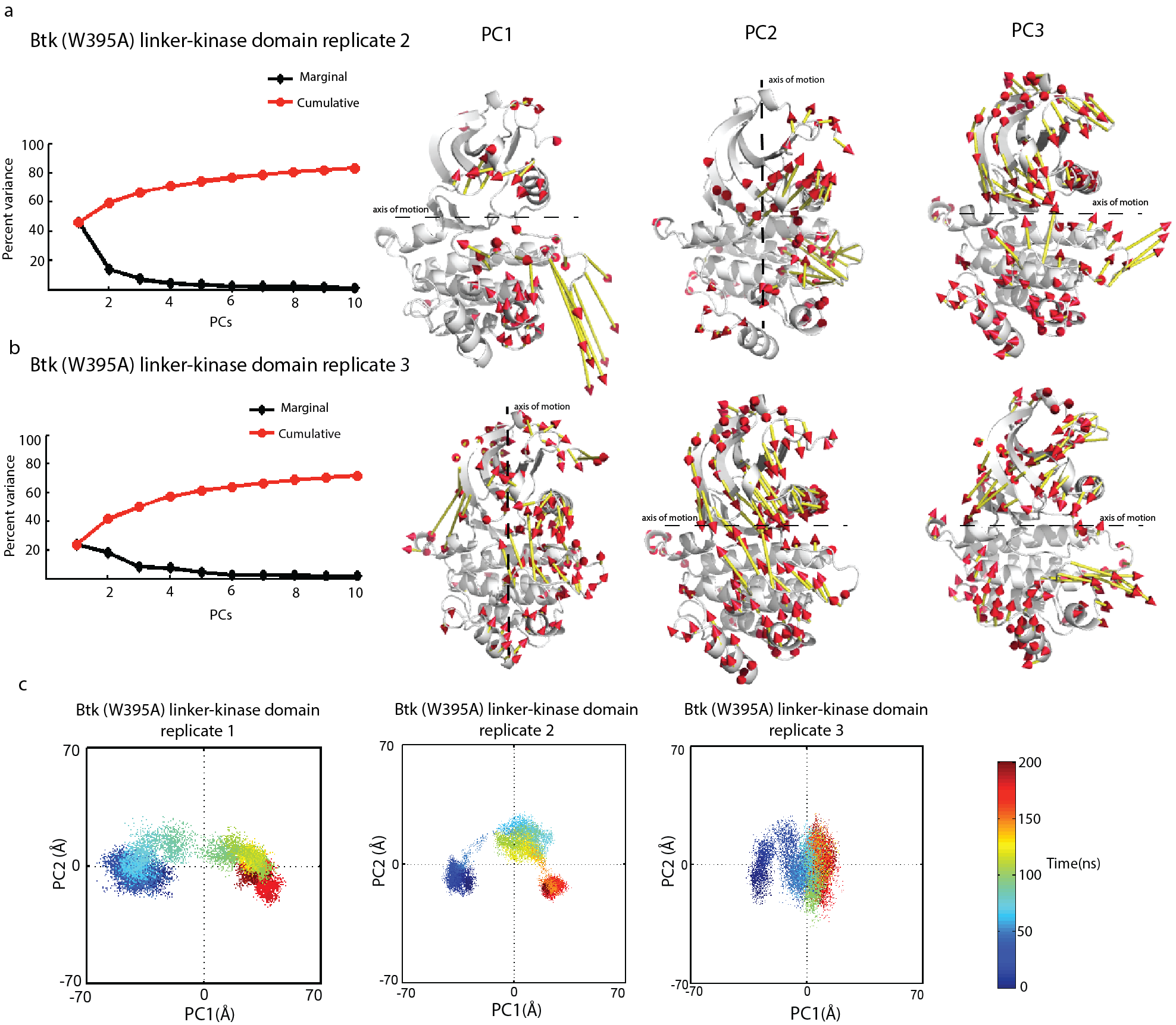
***

**Fig C PCA : Btk (W395A) linker-kinase domain**

Percentage of variance captured by the first 10 Principal Components (PCs) in replicate 2 (a) and replicate 3 (b) for the Btk (W395A) linker-kinase domain. The red line is the cumulative variance captured by the PCs and the black line is the percentage of the variance captured by each individual PC. Directions of motion for the first three PCs for the second (a) and third replicate (b) of Btk (W395A) linker-kinase are shown alongside the percent variance plot. The length of the vectors show the relative magnitudes and the arrowheads indicate the direction of motion. (c) The PC scatter plots showing the projections of the conformations along PC1 and PC2 space, for the first, second and third replicates of Btk (W395A) linker-kinase domain are shown. The color bar indicates simulation timescale.

******

**
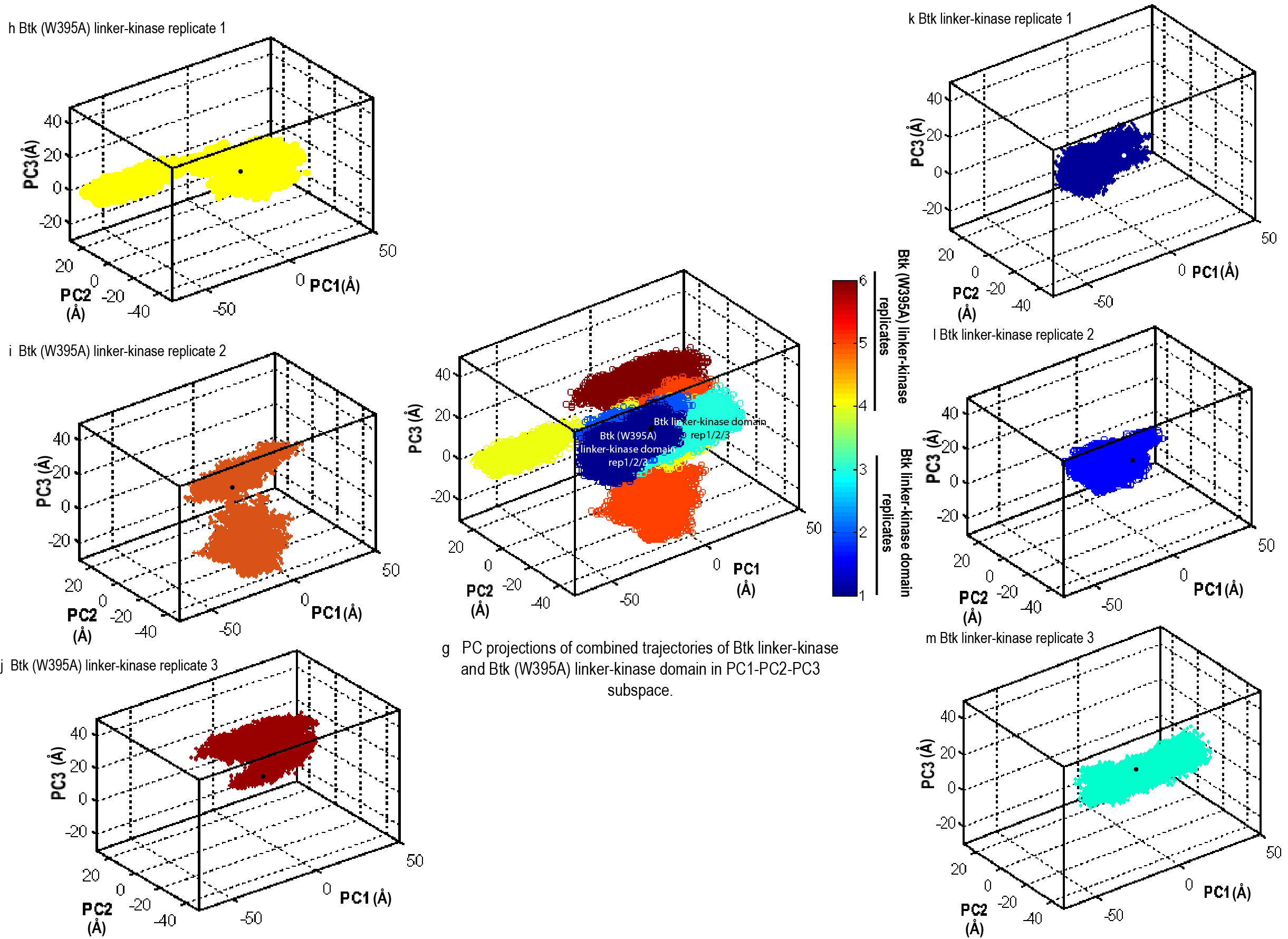
**

**Fig D Overlap of PCs**

Overlap between the first 10 PCs for replicate 1 and 2 (a) replicate 1 and 3 (b) and replicate 2 and 3 (c) for Btk linker-kinase domain simulation. Overlap between the first 10 PCs for replicate 1 and 2 (d) replicate 1 and 3 (e) and replicate 2 and 3 (f) for Btk (W395A) linker-kinase domain simulation. The grey bar indicates the degree of overlap.

**PC scatter plot of combined trajectories of Btk linker-kinase and Btk (W395A) linker-kinase domain in the same subspace.**

Projections of PC scores derived from combining all the simulation replicates of Btk linker-kinase and Btk (W395A) linker-kinase domain in the PC1-PC2-PC3 subspace are shown in (g). The projections of replicates 1, 2, 3 of Btk (W395A) linker-kinase domain in PC1-PC2-PC3 subspace are shown in (h), (i), (j) respectively. The projections of replicates 1, 2, 3 of Btk linker-kinase domain in PC1-PC2-PC3 subspace are shown in (k), (l), (m) respectively. The black filled circle in each scatter plot indicates the starting structure of the simulation trajectory. (In (k) the starting structure is indicated with a white circle.) The colorbar indicates the simulation replicates of Btk linker-kinase and Btk (W395A) linker-kinase domain.

**
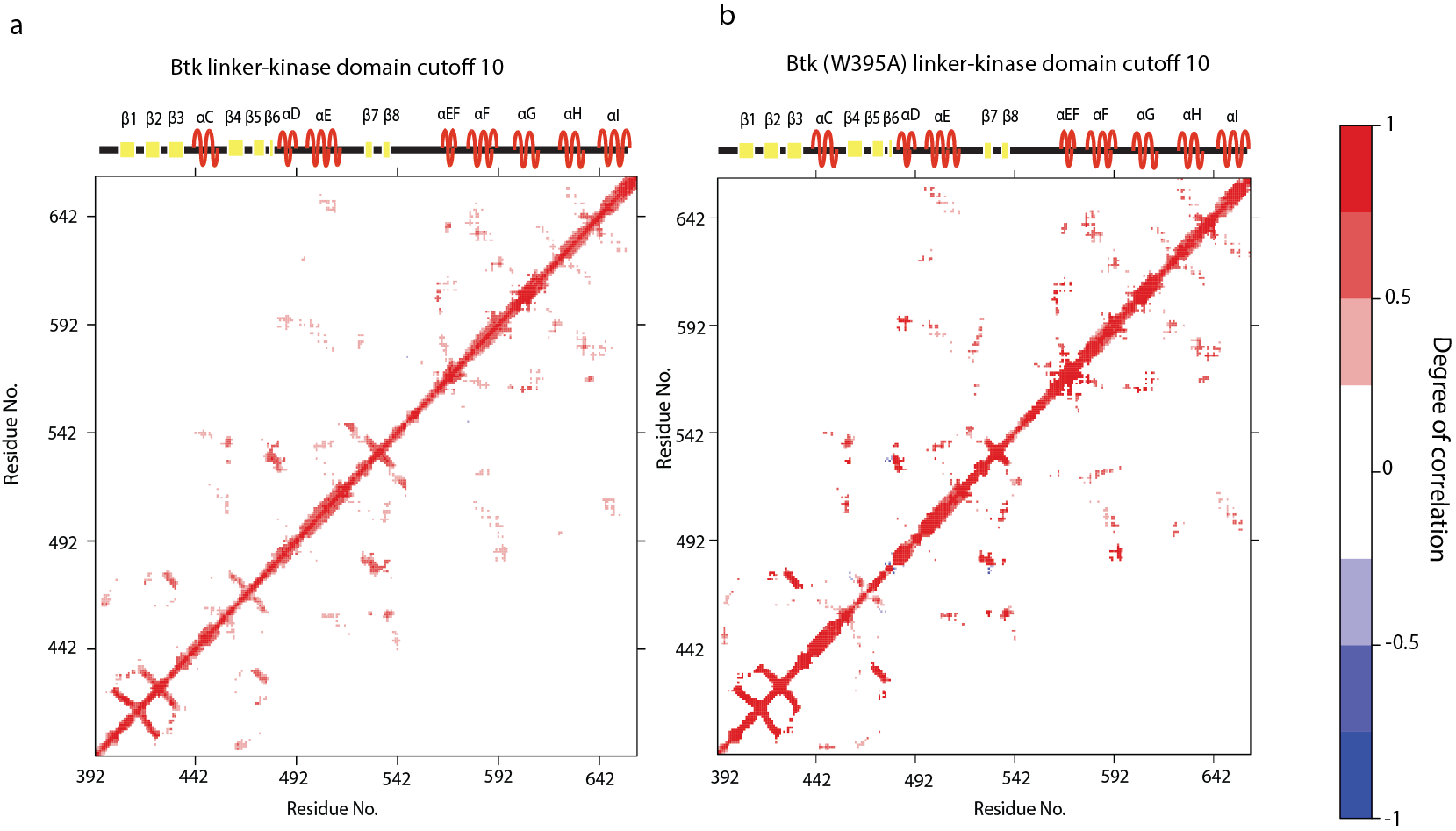
**

**Fig E Dynamic Cross-Correlation maps: Btk linker-kinase and Btk (W395A) linker-kinase domains.**

Covariance matrix of Cα atom pairs within 10 Å in the Btk linker-kinase domain (a) and Btk (W395A) linker-kinase domain (b) represented by the Dynamic Cross-correlation map (DCCM). The color bar indicates the degree of correlation. Red indicates large positive correlations and blue large negative correlations. The secondary structural elements in the Btk kinase domain are shown above each DCCM.

**
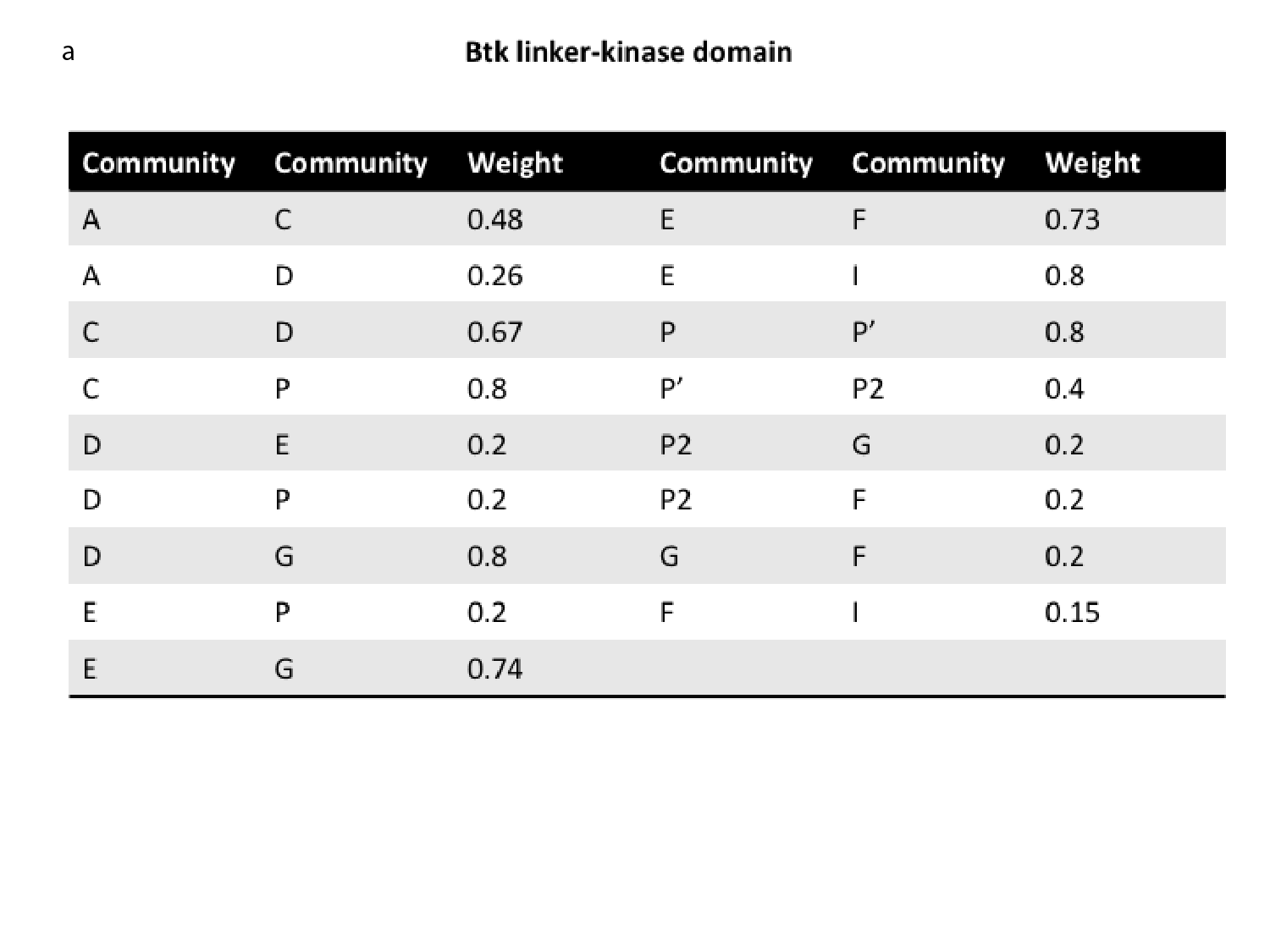
**

***
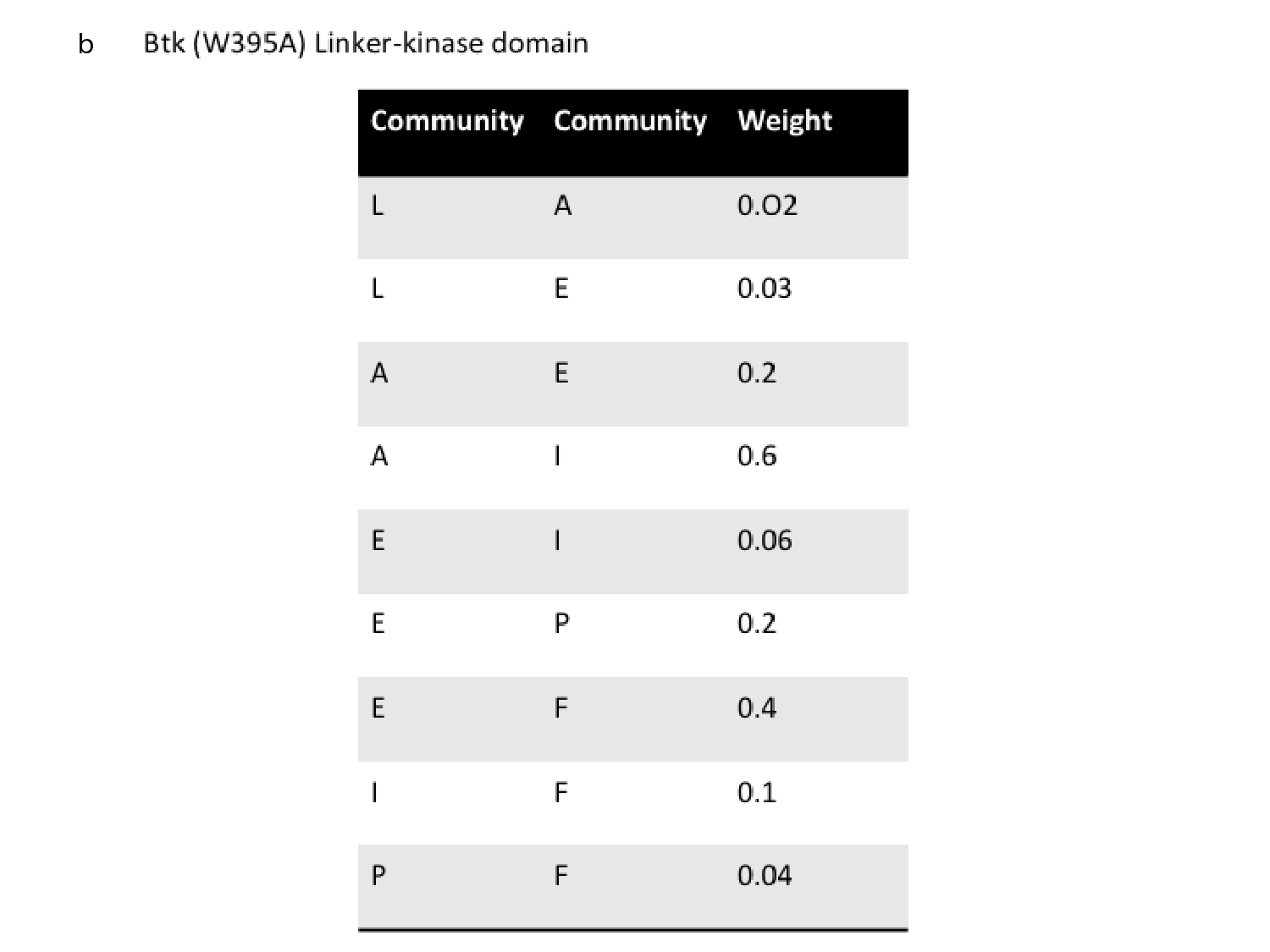
***

**Fig F Edge weights between communities**

Weights of the edges between communities from community analysis of Btk linker-kinase domain (a). Weights of the edges between communities from community analysis of Btk (W395A) linker-kinase domain (b)

**Community Analysis: wild type Btk linker-kinase domain**

Fig G Community Analysis *ComA* *(red)* in Btk linker-kinase domain consists of W395, the Regulatory spine (R-spine) residue L460, Catalytic spine (C-spine) residues V416 and A428 and the gatekeeper residue T474 as well as the bridging residue Y476. It extends from residues 392-431 and 459-476. For all communities, the complete kinase domain is shown on the left and a close-up of the specific community is shown on the right. These residues constitute the beta strands β1, β2, β3, β4, β5, and the Gly-rich loop. *ComA* is involved in positioning ATP in the active site and coordinating dynamics among the structural elements of the N-lobe. *ComA* also contains K430, which participates in a critical salt-bridge with E445 present on the αC-helix. W395 presence in *ComA* signifies its role in the active site arrangement in Btk linker-kinase domain.

Fig G *ComC (yellow)* is localized on the αC-helix and constitutes residues 432-452. It includes the R-spine residue M449. αC-helix is a critical regulatory unit, whose motion from the ‘C-in’ to the ‘C-out’ state mediates the status of the K430-E445 salt-bridge. The αC-helix is found to be a signal integration unit as its motion is coordinated with other critical structural units and thus promotes transmission of allostery to the active site from distant regions of the kinase domain.

Fig G *ComD (green)* consists of the C-spine residues L482, C527 and L528 as well as the proposed bridging residue M477 and L522. It includes residues 453-458, 477-493 from the αD-helix as well as residues 521-539.

Fig G *ComE (dark grey)* is localized on the αE-helix. It includes residues from 494-511.

Fig G *ComP (pink)* includes the R-spine residues H519 and F540. It includes residues from the catalytic loop 512-520 as well as residues from the activation segment N-terminus 540-545.

Fig G *ComP’ (cyan)* consists of residues from the C-terminus of the activation loop (546-555).

Fig G *ComP2 (purple)* consists of residues from the c-terminus of activation segment extending from 556-561, αEF-helix residues 563-575 and 636-637.

Fig G *ComG (magenta)* is localized on the αG-helix and the c-terminus of the αF-helix. It includes residue 562, 586-622. It includes the C-spine residues L586 and I590.

Fig G *ComF (orange)* consists of R-spine reside D579 and the proposed bridging residue F583. It includes residues 576-585, 623-635, 638-644 and 646-647.

Fig G *ComI (pale orange)* is localized on the αI-helix. It includes residue 645, 648-659.

***Community Analysis: Btk (W395A) linker-kinase domain***

Fig G *ComL (dark blue)* consists of linker residues, which includes the A395 mutation as well as residues from the αC-helix, including the salt-bridge forming E445. It extends from residues 392-396, 433-447 as well as 450.

Fig G *ComA (red)* includes the gatekeeper residue T474, salt-bridge forming residue K430, C-spine residues V416 and A428 as well as the high centrality residue Y476. It includes residues from 397-432, 461-477 as well as residue 479.

Fig G *ComE (dark grey)* includes R-spine residues M449, L460, H519 and F540 as well as C-spine residues C527 and L528. It includes residues 448-449, 451-460, 504-523, 526-543.

Fig G *ComP (pink)* is centered on the activation loop residues. It includes residues 544-550.

Fig G *ComF (orange)* is localized on the αF-helix, αG-helix, αEF-helix, the c-terminus of the activation segment and the αH-helix. It includes the R-spine residue D579. It includes residues extending from 551-585, 596-617 and 631-647.

Fig G *ComI (pale orange)* is centered on the αI-helix and includes residues from αD, αE, αF-helices. It includes the C-spine residues L482, L586 and I590 as well as the high centrality residue G480. It includes residues 480-503, 524-525, 586-595, 618-630, and 648-659.
